# Ross procedure versus pulmonary homograft versus mechanical valve versus bioprosthetic valve versus Ozaki procedure for surgical aortic valve replacement: a frequentist network meta-analysis

**DOI:** 10.1186/s43044-023-00391-0

**Published:** 2023-07-22

**Authors:** Ahmed K. Awad, Dave M. Mathew, Peter J. Fusco, Kathryn S. Varghese, Omar Abdel-Nasser, Ayman K. Awad, Peter Giannaris, Serena M. Mathew, Adham Ahmed

**Affiliations:** 1 Faculty of Medicine, Ain Shams University, Cairo, Egypt; 2 City University of New York School of Medicine, 1589 Amsterdam Avenue, New York, NY 10031 USA; 3 Faculty of Medicine, El-Galala University, Suez, Egypt

**Keywords:** Ross procedure, Mechanical valve, Homograft, Aortic valve replacement

## Abstract

**Background:**

There has been a resurgence in interest regarding the Ross procedure due to recent publications detailing positive long-term outcomes. Conversely, surgical aortic valve replacement (SAVR) with a pulmonary homograft (PH), mechanical (MV), bioprosthetic (BV), or the Ozaki procedure each has its own technical advantages and disadvantages. Therefore, we performed a network meta-analysis (NMA) comparing other alternatives to Ross procedure.

**Methods:**

Medical databases were comprehensively searched for studies comparing the Ross procedure with AVR using a PH, MV, BV, or the Ozaki procedure. Outcomes were pooled as risk ratios (RR) with their 95% confidence intervals (95% CI).

**Results:**

A total of 7816 patients were pooled for our NMA from 24 studies. Compared to Ross procedure, both BV and MV were associated with significantly higher rates of 30-day mortality of RR (2.37, 95% CI 1.20–4.67) and (1.88 95% CI 1.04–3.40), respectively, with no significant difference regarding PH or Ozaki. However, only MV was associated with a higher risk of 30-day stroke (RR 8.42, 95% CI 1.57–45.23) with no significant difference in the other alternatives, as well as 30-day MI which showed no significant differences between any of the aortic conduits compared to the Ross procedure. Regarding 30-day major bleeding, MV was associated with a higher when compared to the Ross procedure RR (4.58, 95% CI 1.94–10.85), PH was associated with a lower risk of major bleeding with RR (0.35, 95% CI 0.17–0.71), and BV showed no significant difference. With a mean follow-up duration of 8.5 years compared to the Ross procedure, BV, PH, and MV were associated with a higher risk of long-term mortality with RR (1.89, 95% CI 1.38–2.58), (1.38, 95% CI 1.0–1.87), and (1.94, 95% CI 1.52–2.47), respectively, with the Ozaki procedure showed no significant difference. Regarding long-term stroke—with a mean of 6.3-year follow-up duration—there were no significant differences between any of the aortic conduits compared to the Ross procedure. Nevertheless, long-term need for reintervention—with a mean follow-up duration of 17.5 years—was significant of higher risk with both BV and PH with RR (3.28, 95% CI 1.21–8.84) and (2.42, 95% CI 1.05–5.58), respectively, compared to Ross procedure with MV and Ozaki having no significant difference.

**Conclusions:**

The Ross procedure is a viable treatment option for patients undergoing SAVR, showing promising outcomes at short- and long-term follow-ups.

**Supplementary Information:**

The online version contains supplementary material available at 10.1186/s43044-023-00391-0.

## Background

With aortic valve replacement being the most effective treatment for patients with severe symptomatic aortic valve diseases, determining the ideal conduit for long-term survival and durability has remained elusive. While mechanical valves are known historically to offer excellent durability, the need for lifelong anticoagulation makes them a difficult choice among young patients who are more likely to be nonadherent with strict international normalized ratio (INR) monitoring [1]. Alternatively, bioprostheses do not require lifetime anticoagulation, making them a popular choice for patients hoping to avoid blood thinners, although some operators fear their long-term durability [2, 3]. Of note, the Ross procedure, first described by Dr. Donald Ross in 1967, is a heart valve replacement operation in which the aortic valve is replaced by a pulmonary autograft while substituting the pulmonary position with a homograft [4]. The use of this autologous conduit, alleviates the need for anticoagulants and has been previously shown to offer long-term viability, with a similar hemodynamic profile to the native aortic valve [5, 6].

Many studies have been published in the past advocating for the Ross procedure in patients with long life expectancies [7, 8]. Despite this, Ross procedures have declined in the past two decades due to the technical complexity involved. In addition, the procedure relies on only one surgeon's expertise [9, 10], despite the increased surgical risk and rising rates of autograft failure requiring reintervention [11, 12], Further, new surgical alternatives to mechanical, bioprosthetic, and autograft valves have been proposed, including homograft valves derived from deceased donors or the Ozaki procedure [13]. Aortic valve neocuspidization, or the Ozaki procedure, involves shaping the pericardium into an aortic valve contour, but its effectiveness is still debated compared to conventional substitutes [14]. In this network meta-analysis, we aim to further investigate and compare the various aortic valve conduits to autograft implantation, or the Ross procedure, in terms of safety and effectiveness.

## Methods

### Study guidelines

This NMA was conducted in accordance with the Meta-Analysis of Observational Studies in Epidemiology (MOOSE) [15] (Additional file 1: Figure 1) and Preferred Reporting Items for Systematic Reviews and Meta-Analyses (PRISMA) [16] guidelines (Additional file 1: Figure 2).

### Comprehensive literature search strategy

A comprehensive literature search was performed on the PubMed, Scopus, Web of Science, Embase, and Cochrane Library libraries to identify observational cohort studies and randomized control trials (RCT) studies comparing the use of the Ross pulmonary autograft procedure versus the use of pulmonary homograft, aortic neocuspidization (Ozaki) conduit, mechanical, and/or bioprosthetic valve for aortic valve replacement. The libraries were searched without restrictions from inception to October 1, 2022. Search terms included ‘aortic valve replacement’, ‘surgical aortic valve replacement’, ‘Ross procedure’, ‘switch procedure’, ‘pulmonary autograft procedure’, ‘autograft valve’, ‘homograft valve’, ‘mechanical aortic valve replacement’, Ozaki procedure’, ‘aortic valve neocuspidization’, and ‘bioprosthetic aortic valve replacement’ (Additional file 1: Table 1). Additionally, the references of the retrieved studies were searched for relevant papers.

### Inclusion/exclusion criteria

Studies providing comparative data between at least one specific aortic conduit with autograft or the Ross procedure were eligible for analysis. Studies that combined or studied homograft valve (HV), mechanical valve (MV), bioprosthetic valve (BV), or the Ozaki procedure into a singular treatment arm and/or those that did not clearly define their valve type were excluded. Additionally, editorials, single-arm studies, review articles, and conference presentations, were ineligible for analysis.

### Study selection/data extraction

The final search strategy identified 1,813 studies. After the removal of duplicates, two investigators (S.M. & S.K.) independently screened 1,545 citations based on title/abstract. Following the primary screening, two authors (K.V. & A.S.) reviewed the full texts of the remaining articles for eligibility. Any disagreements during screening were resolved by discussion with a third author (A.K.A.). Our screening process is summarized via the PRISMA flow diagram available in Additional file 1: Figure 2.

Two independent investigators (K.V. & D.M.) performed data extraction studies; with a third reviewer (A.A.) for accuracy. The following were extracted from each article: study information (author names/affiliation, year of publication, duration of care, number of patients in each group) (Additional file 1: Table 2), baseline characteristics (age, gender, EuroSCORE, STS risk score, NYHA Class III/IV, BMI, presenting etiology (aortic stenosis/ regurgitation), hypertension, hypercholesterolemia, prior stroke, prior myocardial infarction, diabetes, left ventricular disease, tobacco use, peripheral vessel disease, coronary artery disease, chronic obstructive pulmonary disease, kidney failure, heart failure, previous prior cardiac surgeries, prior pacemaker, and atrial fibrillation) (Additional file 1: Table 3), operative details [operative time, fluoroscope time, cardiopulmonary bypass, aortic prosthesis size, coronary obstruction, annular rupture, length of ICU stay, length of index hospital stay] (Additional file 1: Table 4), and post-operative outcomes. Our primary outcomes were 30-day mortality, stroke, myocardial infarction, and major bleeding, while secondary outcomes were long-term mortality, stroke, and risk of reintervention.

### Study quality assessment

For observational cohort studies, quality was determined using the Newcastle Ottawa Scale [17] (Additional file 1: Table 5). Conversely, RCT quality was assessed using the Cochrane Collaboration’s tool for assessing the risk of bias, version 2, in randomized trials [18] (Additional file 1: Table 6). Two authors (K.V. & D.M.) independently judged study quality, with discrepancies being solved by discussion to reach a consensus.

### Statistical analysis

**Fig. 1:**
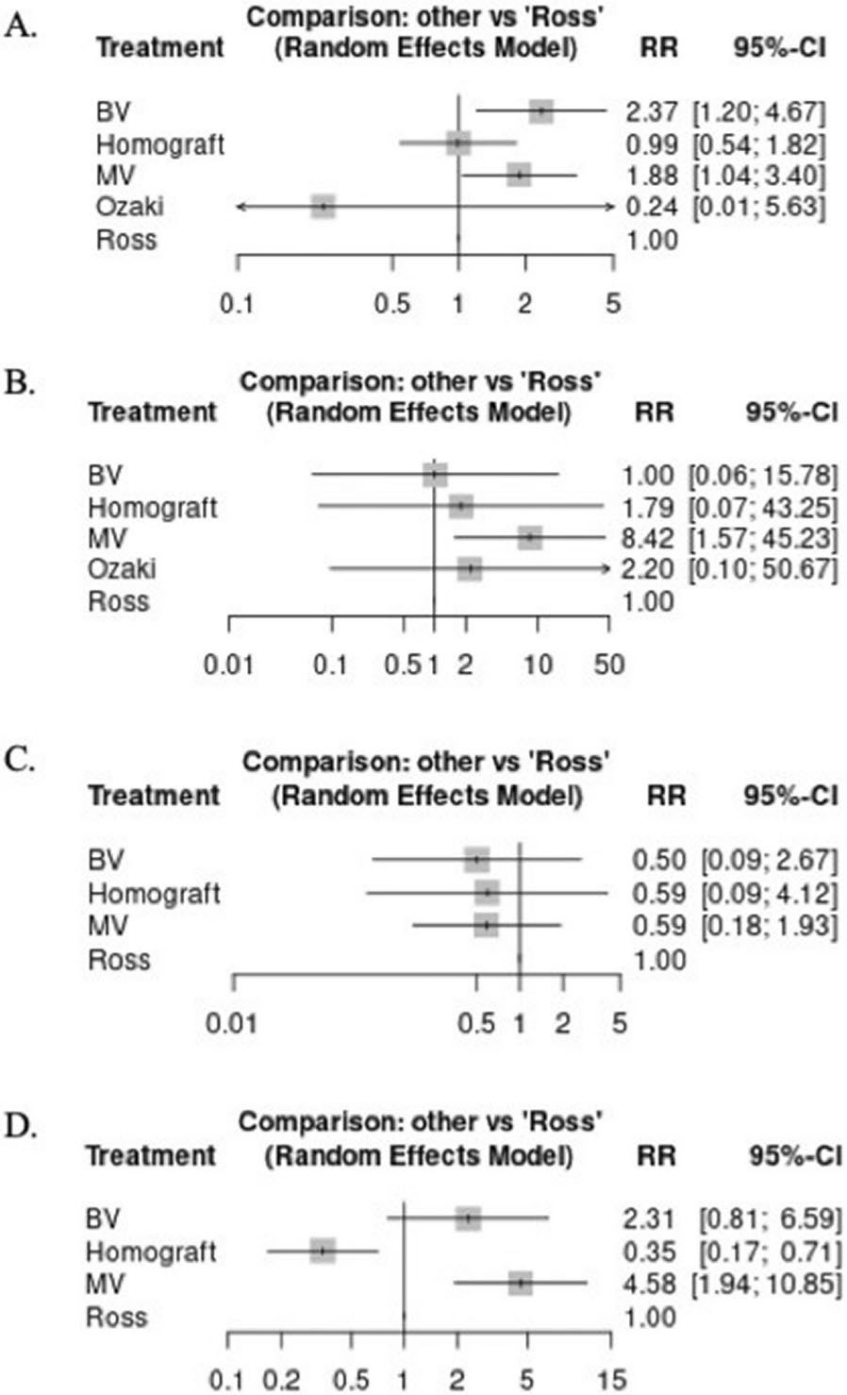
Forest plot comparing bioprosthetic valve (BV), homograft valve, mechanical valve (MV), Ozaki, and Ross for **A** 30-day mortality **B** 30-day stroke **C** 30-day myocardial infarction **D** 30-day major bleeding. *RR* risk ratio

**Fig. 2:**
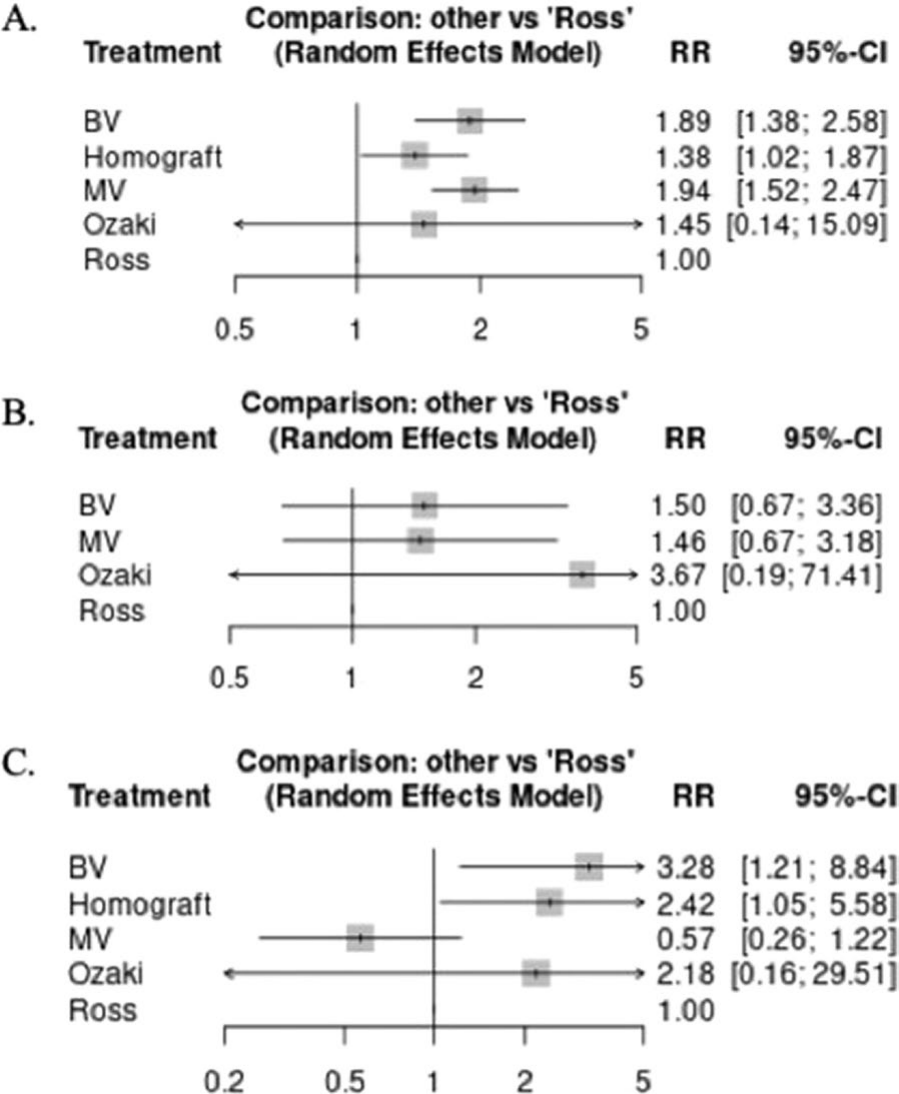
Forest plot comparing bioprosthetic valve (BV), homograft valve, mechanical valve (MV), Ozaki, and Ross for **A** long-term mortality **B** long-term stroke and **C** long-term reintervention. *RR* risk ratio

All outcomes were pooled in the random-effects model as risk ratios (RR) with 95% confidence intervals (CIs). Frequentist network meta-analysis (NMA) was performed through the Meta-Insight software v1.1 [19]. Heterogeneity between the studies was assessed using The Higgins’ and Thompson’s I^2^ statistic^20^, and rated as low (*I*^2^ = 0–25%), moderate (*I*^2^ = 25–50%) or high (*I*^2^ > 50%) (Figs. 1, 2, Additional file 1: Figures 3–6). Heat plots were generated, and statistical significance was set at *p* value < 0.05 for all analyses.

## Results

### Baseline characteristics

Our comprehensive search strategy retrieved 1813 studies. After applying inclusion/exclusion criteria, 24 studies were included in our network meta-analysis (Additional file 1: Table 6). Of the included reports, two were RCTs and nine were propensity-matched observational studies. A summary of the included reports can be found in Table 1.

**Table 1:** Summary of the included studies

Author	Study type	Number of patients
Akhyari 2005	Retrospective observational	38 (18 Ross; 20 MV)
Andreas 2014	Retrospective observational	332 (159 Ross; 173 MV)
Bouhout 2017	Prospective observational with PSM	140 (70 Ross; 70 MV)
Buratto 2018	Retrospective observational with PSM	550 (275 Ross; 275 MV)
Choudhary 1998	Retrospective observational	189 (96 Ross; 93 Homograft)
Dagenais 2005	Prospective observational	332 (76 Ross; 202 BV; 54 Homograft)
Doss 2005	RCT	40 (20 Ross; 20 MV)
Doss 2011	Prospective observational	40 (20 Ross; 20 MV)
Elhamamsy 2010	Prospective observational	216 (108 Ross; 108 Homograft)
Elhamamsy 2022	Retrospective observational with PSM	1,302 (434 Ross; 434 MV; 434 BV)
Gofus 2022	Retrospective observational with PSM	582 (291 Ross; 291 MV)
Grocott-Mason 2000	Retrospective observational	518 (90 MV; 47 BV; 381 Homograft)
Heuvelman 2013	Retrospective observational	40 (18 Ross; 9 MV; 13 Homograft)
Jaggers 1998	Prospective observational	49 (22 Ross; 27 MV)
Klieverik 2008	Prospective observational	169 (63 Ross; 106 Homograft)
Knott-Craig 2000	Retrospective observational	238 (145 Ross; 93 Homograft)
Laforest 2002	Prospective observational	243 (132 Ross; 111 Homograft)
Mazine 2016	Retrospective observational with PSM	416 (208 Ross; 208 MV)
Mazine 2022	Retrospective observational with PSM	216 (108 Ross; 108 BV)
Mokhles 2011	Retrospective observational with PSM	506 (253 Ross; 253 MV)
Polito 2021	Retrospective observational	38 (16 Ross; 22 Ozaki)
Santini 1997	RCT	70 (33 Ross; 37 Homograft)
Sharabiani 2016	Retrospective observational with PSM	1,501 (717 Ross; 567 MV; 164 BV; 53 Homograft)
Zacek 2016	Retrospective observational	51 (22 Ross; 29 MV)

A total of 7816 patients were pooled for NMA: 3304 underwent the Ross procedure, 2486 received mechanical valves, 955 received bioprosthetic valves, 1,049 received homografts, and 22 underwent the Ozaki procedure. The number of patients in each study ranged from 22 to 434, while the mean age ranged from 22.9 to 85.0 years old. Male patients constituted between 26.7 and 69.4% of the study population (Additional file 1: Tables 2–3).

### Quality assessment

Both of our included randomized controlled trials showed a low risk of bias. Furthermore, out of 22 observational studies, 16 was of good quality. Further details about the risk of bias in our studies can be found in Additional file 1: Table 6.

### Short-term outcomes (Fig. 1)

#### 30-day mortality

Compared to the Ross procedure, both BV and MV were associated with significantly higher rates of 30-day mortality (BV: RR 2.37, 95% CI 1.20–4.67; MV: 1.88, 95% CI 1.04–3.40). There was no significant difference in 30-day mortality between homograft or Ozaki and the Ross procedure. As shown in the ranking table (Fig. 1A, Additional file 1: Table 7), the Ozaki procedure ranked first in terms of lower mortality risk, followed by homograft and the Ross procedure. No heterogeneity was observed (*I*^2^ = 0%, *p* = 0.75). Further details about inconsistencies between studies are found in Additional file 1: Figures 3 and 4.

#### 30-day stroke

Compared to the Ross procedure, only MV was associated with a higher risk of 30-day stroke (RR 8.42, 95% CI 1.57–45.23). Conversely, no difference was seen between BV, homograft, and the Ozaki procedure when compared to the Ross procedure. As shown in the ranking table (Fig. 1B, Additional file 1: Table 8), Ross had the lowest stroke risk followed by BV, homograft, and MV. No heterogeneity was observed (*I*^2^ = 0%, *p* value = 0.95). Further details about inconsistencies between studies are found in Additional file 1: Figures 3.1 and 4.1.

#### 30-day myocardial infarction (MI)

There were no significant differences in 30-day MI between any of the aortic conduits compared to the Ross procedure. According to the ranking table (Fig. 1C, Additional file 1: Table 9), BV had the lowest MI risk, followed by MV, homograft, then the Ross procedure. Low heterogeneity was observed (*I*^2^ = 15%, *p* = 0.52). Further details about inconsistencies between studies are found in Additional file 1: Figures 3.2 and 4.2.

#### 30-day major bleeding

MV was associated with a higher risk of 30-day major bleeding when compared to the Ross procedure (RR 4.58, 95% CI 1.94–10.85). Although homograft was associated with a lower risk of major bleeding when compared to the Ross procedure (RR 0.35, 95% CI 0.17–0.71), BV showed no significant difference. According to the ranking table (Fig. 1D, Additional file 1: Table 10), homograft ranked first in terms of lower major bleeding risk, followed by the Ross procedure, BV, and MV. Low heterogeneity was observed (*I*^2^ = 18%, *p* = 0.53).

Further details about inconsistencies between studies are found in Additional file 1: Figures 3.3 and 4.3.

### Long-term outcomes (Fig. 2)

#### Long-term mortality

With mean follow-up duration of 8.5 years compared to the Ross procedure, BV, homograft, and MV were associated with a higher risk of long-term mortality (BV: RR 1.89, 95% CI 1.38–2.58; Homograft: RR 1.38, 95% CI 1.0–1.87; MV: RR 1.94, 95% CI 1.52–2.47). The Ozaki procedure showed no significant difference when compared to the Ross procedure. In terms of lower mortality risk according to rank table (Fig. 2A, Additional file 1: Table 11), the Ross procedure ranked first, followed by homograft and the Ozaki procedure, then BV and MV. Moderate heterogeneity was observed (*I*^2^ = 23%, *p* = 0.58). Further details about inconsistencies between studies are found in Additional file 1: Figures 3.4 and 4.4.

#### Long-term stroke

With mean follow-up duration of 6.3 years, there were no significant differences in long-term stroke between any of the aortic conduits compared to the Ross procedure. As reported in the ranking table (Fig. 2B, Additional file 1: Table 12), the Ross procedure ranked highest, followed by MV, BV, and the Ozaki procedure. Moderate heterogeneity was observed (*I*^2^ = 38%, *p* = 0.25). Further details about inconsistencies between studies found in Additional file 1: Figures 3.5 and 4.5.

#### Long-term reintervention

With mean follow-up duration of 17.5 years compared to the Ross procedure, both BV and homograft were associated with higher rates of long-term need for reintervention (BV: RR 3.28, 95% CI 1.21–8.84; Homograft: RR 2.42, 95% CI 1.05–5.58). MV and Ozaki showed no significant difference when compared to the Ross procedure. According to the ranking table (Fig. 2C, Additional file 1: Table 13), MV ranked first followed by the Ross and Ozaki procedures, then homograft and BV. Low heterogeneity was observed (*I*^2^ = 18%, *p* = 0.412). Further details about inconsistencies between studies are found in Additional file 1: Figures 3.6 and 4.6.

## Discussion

To the best of our knowledge, this network meta-analysis of over 24 studies involving 7500 patients undergoing aortic valve replacement is the most comprehensive comparison of aortic conduits available. Our results showed that compared to the Ross procedure, both BV and MV were associated with significantly higher rates of 30-day mortality, with no difference observed between the Ross, homograft, and Ozaki procedures. Further, MV was associated with a higher risk of 30-day stroke and major bleeding compared to the Ross procedure. Patients receiving BV, MV, and pulmonary homograft had significantly higher long-term mortality rates compared with Ross, along with significantly higher long-term reintervention rates for BV and homograft. No differences were observed for long-term stroke.

Previously, several pooled reports have attempted to assess the safety and efficacy of various forms of aortic valve replacement. According to McClure and colleagues' 2019 systematic review and meta-analysis, with 5,346 patients from 15 studies pooled together [21], the Ross procedure did not result in a significant difference in < 30-day mortality but significantly decreased late-mortality after 2.6 years of follow-up (RR 0.56, 95% CI 0.38–0.84, p = 0.005). Of note, their control group pooled various alternate aortic conduits, including pulmonary homograft, as well as mechanical/tissue prostheses. More recently, a meta-analysis of reconstructed individual participant data by Tasoudis et al. [22] pooled 17,683 patients from 25 studies undergoing aortic valve replacement via MV or BV and reported lower overall mortality in MV (hazard ratio (HR) 0.79, 95% CI 0.74–0.84, *p* < 0.0001).

Notably, a recent network meta-analysis presented at the 2022 American Heart Association conference by Yokoyama et al. [23] sought to compare the efficacy of the Ross procedure compared to aortic valve replacement using MV or BV. Pooling ten comparative reports (2 RCTs, 8 propensity score–matched), the authors found the Ross procedure to be associated with significantly lower all‐cause mortality compared to both artificial valves (MV: HR 0.58, 95% CI 0.35–0.97; *p* = 0.035, BV: HR 0.32, 95% CI 0.18–0.59;  *p* < 0.001). Additionally, the Ross showed lower reintervention rates compared to BV (HR 0.31, 95% CI 0.15–0.65; *p* = 0.002), but a higher need for reintervention compared to MV (HR 2.12, 95% CI 1.04–4.33, *p* = 0.039). Our present report helps build upon the work of Yokoyama et al. by confirming their findings of the Ross procedures’ protectiveness against long-term mortality compared to BV and MV, while also incorporating further comparisons with alternate aortic conduits (protective compared to pulmonary homograft, with no difference compared to Ozaki procedure). Interestingly, our pooled analyses for long-term reintervention showed no significant difference between the Ross procedure and MV.

In line with prior literature, we demonstrated a mortality benefit with the Ross procedure compared to MV and BV at long-term follow-up [24, 25]. Widely praised for its ability to provide comparable post-operative life expectancy to that of the matched general population [26], the pulmonary autograft’s utilization provides patients with a conduit capable of adaptive remodeling to the mobile native aortic root and preservation of native hemodynamic and contractile properties [27]. Also of note, when deeply positioning the autograft within the left ventricular outflow tract, the Ross procedure helps confer a lower risk of severe patient-prosthesis mismatch, which carries a higher risk of morbidity and mortality [28]. Conversely, prosthetic valves, particularly those with obstructive stented leaflets may increase the risk of improper sizing and malpositioning. [25, 29]

Two notable findings in our pooled Ross versus MV comparisons were an increased risk for 30-day stroke and 30-day major bleeding with the use of a mechanical conduit. Previously, several reports have reported a higher risk for cerebrovascular and hemorrhagic events following MV aortic valve replacement compared to the pulmonary autograft. According to Mazine and colleagues' pairwise meta-analysis of 3,516 patients from 18 studies comparing the Ross procedure with MV, the Ross procedure is associated with a lower stroke incidence (IRR 0.26, 95% CI 0.09–0.80, *p* = 0.02) and major bleeding rate (IRR 0.17, 95% CI 0.07–0.40, *p* < 0.001) [30]. More recently, a multicenter propensity-matched registry analysis by El-Hamamsy et al. [29] found that patients undergoing the Ross procedure had a lower 15-year cumulative incidence of stroke (2.1% v 4.8%, *p* = 0.03) and major bleeding (1.9% vs 5.2%, *p* = 0.016). These findings may be a consequence of MV necessitation of anticoagulation therapy and subsequent dosing and nonadherence complications. The use of a lower INR ratio following mechanical valve replacement remains a point of ongoing research. [31, 32]

With regard to the pulmonary homograft, we reported higher rates of long-term mortality and reintervention compared to the Ross procedure. Plausible explanations of this include the homograft’s inferior hemodynamic performance, and a higher risk of patient-prosthesis mismatch and premature degeneration, particularly among younger patients [33, 34]. Based on prior literature, our findings are consistent with prior comparisons of homografts to autografts; El-Hamamsy and colleagues published a randomized control trial in the Lancet in 2010 that found pulmonary homograft-operated patients were more likely to die after ten years compared with Ross-operated patients (HR 4.61, 95% CI 1.71–16.03, *p* = 0.0060), and homograft was an independent predictor of reoperation on multivariate analysis (HR 5.69, 95% CI 2.46–13.15) [35]. Similarly, Etnel et al. reported higher reintervention rates in children that underwent aortic valve replacement using a pulmonary homograft compared to the Ross autograft (5.44%/year, 95% CI 4.24–6.98). [36]

Notably, we found no significant difference between the Ross and Ozaki procedures, with the latter even ranking first in the prevention of 30-day mortality. In recent years, aortic valve neocuspidization via the Ozaki procedure has grown as a viable conduit option for pediatric aortic valve replacement. Unfortunately, comparative clinical literature on the procedure remains limited with the only report assessing its efficacy to our knowledge being a 2021 retrospective study of 38 patients by Polito et al. showing similar rates of mortality and reoperation at mid-term follow-up [37]. Benefits of this technique include the preservation of aortic root anatomy and cardiac cycle synchronization without necessitating a pulmonic trunk explantation and allograft implantation. Notably, at short-term assessment, cardiovascular magnetic resonance studies have shown similar rates of proximal aortic wall shear stress values with the Ozaki procedure and the Ross method [38]. Additionally, similar to a pulmonary autograft, aortic neocuspidization precludes the need for life-long anticoagulation therapy with subsequent risk of bleeding and thrombotic events [37]. Further prospective data will be crucial in ascertaining the long-term performance of the Ozaki procedure compared to the Ross and other aortic valve conduits.

Looking forward, a multidisciplinary review of patient etiology, aortic root anatomy, comorbidities, and institutional expertise/resources will always be crucial to optimize conduit selection for aortic valve replacement. Our network meta-analysis supports the Ross procedure as a viable option for patients, showing non-inferior to superior outcomes for all explored outcomes compared to pulmonary homograft, BV, MV, and the Ozaki procedure. It is worth highlighting that a large concern for many operators, and patients is the creation of a “bi-valvular disorder,” with a heightened risk of reoperation on either the autograft aortic or allograft pulmonic valves. According to many contemporary reports, reintervention risk is often operator-dependent, and the optimization of surgical techniques and appropriate valve sizing can help reduce the risk of reintervention. The use of decellularized pulmonary homografts and trimming excess autograft and supracommisural pulmonary artery tissue, deep intra-annular autograft positioning, and deep intra-annular autograft positioning at the initial intervention facilitate longer periods without reoperation [24].

Our NMA has several pertinent limitations that warrant consideration. First, 15 of our pooled reports were retrospective in nature, which comes with the risk of operator and treatment allocation biases. To help account for this, we predetermined the use of a random effects model which helps control for inter-study heterogeneity and prioritized propensity-matched subgroup data whenever provided by report. Next, a relatively low number of studies provided outcome data for 30-day and long-term stroke, which may make it difficult for us to make sound conclusions. Readership must interpret out analysis with such limitation in mind and note that our sample size may have been underpowered to detect additional difference in stroke incidence. Similarly, there was a scarcity of literature reporting outcomes on the Ozaki procedure compared to the Ross procedure, limiting this treatment arm to only one report with 22 patients in our network meta-analysis. While we found it important to include this report in our analysis, it is evident that there is a large gap in the literature when it comes to the Ozaki procedure compared to more traditional forms of aortic valve replacement. Additionally, since our report utilizes the Ross procedure as the reference arm, we restricted our search strategy to exclude studies that did not include a Ross comparison arm for our reference arm. This hindered our ability to build a substantive direct/indirect evidence network for outcomes such as 30-day myocardial infarction where fewer direct Ross versus alternate arm comparisons existed in the literature. Finally, our report depended on study-level data as we could not access individual patient characteristics. This limited our ability to perform pertinent subgroup analyses, such as by age group and also made it difficult for us to assess additional important metrics of assessing conduit success, such as echocardiographic performance and need for pacemakers. Also of note, our comprehensive literature search included only full reports published up to October 2022 and it is possible that we missed manuscripts published past this date or manuscripts that have yet to be published, such conference papers showcasing preliminary data [40, 41]. Looking forward, future multicenter prospective studies may be helpful to ascertain the efficacy of these techniques stratified across various patient age groups and surgical risk factors.

## Conclusions

Compared to the Ross procedure, BV and MV were associated with higher risks of both 30-day and long-term mortality, with MV showing an increased risk for both 30-day stroke and major bleeding. Moreover, homograft was associated with higher risks for both long-term mortality and stroke when compared to the Ross procedure. In terms of 30-day myocardial infarction and long-term stroke, there was no difference between any aortic conduit and the Ross procedure.

## Supplementary Information

**Additional file 1.** Supplemental Material including supplemental figures, tables, and references.

## Data Availability

All data generated or analyzed during this study are included in this published article [and its additional file 1].

## Abbreviations

PH: Pulmonary homograft
MV: Mechanical valve
BV: Bioprosthetic valve
CI: Confidence interval
RR: Risk ratio
NMA: Network meta-analysis
