# Supplementary material for: Ross procedure versus pulmonary homograft versus mechanical valve versus bioprosthetic valve versus Ozaki procedure for surgical aortic valve replacement: a frequentist network meta-analysis
Source: Egypt Heart J. 2023 Jul 22;75:64. doi: 10.1186/s43044-023-00391-0 (PMC10361932; doi:10.1186/s43044-023-00391-0)
Supplement: Supplementary file 1 — Additional file 1. Supplemental Material including supplemental figures, tables, and references. [file 43044_2023_391_MOESM1_ESM.docx]

**APPENDIX**

**Supplementary Figure 1.** The Meta-Analysis of Observational Studies in Epidemiology (MOOSE) Reporting checklist for our analysis.

**Supplementary Figure 2.** Preferred Reporting Items for Systematic Reviews and Meta-Analysis (PRISMA) flowchart of our analysis.

**Supplementary Figure 3.** Individual study results (for all studies) grouped by treatment comparison for 30-day mortality.

**Supplementary Figure 3.1.** Individual study results (for all studies) grouped by treatment comparison for 30-day stroke.

**Supplementary Figure 3.2.** Individual study results (for all studies) grouped by treatment comparison for 30-day myocardial infarction.

**Supplementary Figure 3.3.** Individual study results (for all studies) grouped by treatment comparison for 30-day major bleeding.

**Supplementary Figure 3.4.** Individual study results (for all studies) grouped by treatment comparison for long-term mortality.

**Supplementary Figure 3.5.** Individual study results (for all studies) grouped by treatment comparison for long-term stroke.

**Supplementary Figure 3.6.** Individual study results (for all studies) grouped by treatment comparison for long-term reintervention.

**Supplementary Figure 4.** Network plot of all studies for 30-day mortality.

**Supplementary Figure 4.1.** Network plot of all studies for 30-day stroke.

**Supplementary Figure 4.2.** Network plot of all studies for 30-day myocardial infarction.

**Supplementary Figure 4.3.** Network plot of all studies for 30-day major bleeding.

**Supplementary Figure 4.4.** Network plot of all studies for long-term mortality.

**Supplementary Figure 4.5.** Network plot of all studies for long-term stroke.

**Supplementary Figure 4.6.** Network plot of all studies for long-term reintervention.

**Supplementary Table 1.** Search strategy used and applied on different databases

**Supplementary Table 2.** Summary of the included studies.

**Supplementary Table 3.** Patient demographics, risk factors, and comorbidities of the included studies.

**Supplementary Table 4.** Operative details of the included studies.

**Supplementary Table 5.** Summary of critical appraisal of included observational studies using the Newcastle Ottawa Quality Assessment Scale for Cohort Studies.

**Supplementary Table 6.** The Cochrane Risk-of-Bias Tool for Randomized Trials (RoB 2).

**Supplementary Table 7.** Rank table of outcome 30 days mortality

**Supplementary Table 8.** Rank table of outcome 30 days stroke

**Supplementary Table 9.** Rank table of outcome 30 days MI

**Supplementary Table 10.** Rank table of outcome 30 days major bleeding

**Supplementary Table 11.** Rank table of outcome long term mortality

**Supplementary Table 12.** Rank table of outcome long term stroke

**Supplementary Table 13.** Rank table of outcome long term re-intervention

**Supplementary Table 14.** Summary of our outcomes

**Supplementary Table 15.** References of Included Studies.

This supplementary material has been provided by the authors to give readers additional information about their work.

**Supplementary Figure 1.** The Meta-Analysis of Observational Studies in Epidemiology (MOOSE) Reporting checklist for our analysis.

| **Reporting Criteria** | **Reported (Yes/No)** | **Reported on Page** |
| --- | --- | --- |
| **Reporting of Background** |  |  |
| Problem definition | Yes | 4 |
| Hypothesis statement | Yes | 4 |
| Description of Study Outcome(s) | Yes | 8 |
| Type of exposure or intervention used | Yes | 4 |
| Type of study design used | Yes | 4 |
| Study population | Yes | 4 |
| **Reporting of Search Strategy** |  |  |
| Qualifications of searchers (eg, librarians  and investigators) | No |  |
| Search strategy, including time period  included in the synthesis and keywords | Yes | 5 |
| Effort to include all available studies,  including contact with authors | No |  |
| Databases and registries searched | Yes | 5 |
| Search software used, name and  version, including special features used  (eg, explosion) | No |  |
| Use of hand searching (eg, reference  lists of obtained articles) | Yes | 5 |
| List of citations located and those  excluded, including justification | Yes | 5-6 |
| Method for addressing articles  published in languages other than  English | Yes | 5 |
| Method of handling abstracts and  unpublished studies | Yes | 6 |
| Description of any contact with authors | No |  |
| **Reporting of Methods** |  |  |
| Description of relevance or  appropriateness of studies assembled for  assessing the hypothesis to be tested | Yes | 6 |
| Rationale for the selection and coding of  data (eg, sound clinical principles or  convenience) | Yes | 5-6 |
| Documentation of how data were  classified and coded (eg, multiple raters,  blinding, and interrater reliability) | Yes | 5-6 |
| Assessment of confounding (eg,  comparability of cases and controls in  studies where appropriate | No |  |
| Assessment of study quality, including  blinding of quality assessors;  stratification or regression on possible  predictors of study results | Yes | 9 |
| Assessment of heterogeneity | Yes | 9 |
| Description of statistical methods (eg,  complete description of fixed or random  effects models, justification of whether  the chosen models account for predictors  of study results, dose-response models,  or cumulative meta-analysis) in sufficient  detail to be replicated | Yes | 8-9 |
| Provision of appropriate tables and  graphics | Yes | 8-9 |
| **Reporting of Results** |  |  |
| Table giving descriptive information for  each study included | Yes | 7-8 |
| Results of sensitivity testing (eg,  subgroup analysis) | Yes | 9 |
| Indication of statistical uncertainty of  findings | Yes | 9 |
| **Reporting of Discussion** |  |  |
| Quantitative assessment of bias (eg,  publication bias) | Yes | 9 |
| Justification for exclusion (eg, exclusion  of non–English-language citations) | Yes | 5-6 |
| Assessment of quality of included studies | Yes | 6 |
| **Reporting of Conclusions** |  |  |
| Consideration of alternative explanations  for observed results | Yes | 15-16 |
| Generalization of the conclusions (ie,  appropriate for the data presented and  within the domain of the literature review) | Yes | 17 |
| Guidelines for future research | Yes | 16 |
| Disclosure of funding source | Yes | 19 |

**Supplementary Figure 2.** Preferred Reporting Items for Systematic Reviews and Meta-Analysis (PRISMA) NMA Checklist of Items to Include When Reporting A Systematic Review Involving a Network Meta-analysis (NMA)

| **Section/Topic** | **Item #** | **Checklist Item** | **Reported on Page #** |
| --- | --- | --- | --- |
| **TITLE** |  |  |  |
| Title | 1 | Identify the report as a systematic review *incorporating a network meta-analysis (or related form of meta-analysis).* | ***Title*** |
|  |  |  |  |
| **ABSTRACT** |  |  |  |
| Structured summary | 2 | Provide a structured summary including, as applicable:  **Background:** main objectives  **Methods:** data sources; study eligibility criteria, participants, and interventions; study appraisal; and *synthesis methods, such as network meta-analysis.*  **Results:** number of studies and participants identified; summary estimates with corresponding confidence/credible intervals; *treatment rankings may also be discussed. Authors may choose to summarize pairwise comparisons against a chosen treatment included in their analyses for brevity.*  **Discussion/Conclusions:** limitations; conclusions and implications of findings.  **Other:** primary source of funding; systematic review registration number with registry name. | 1 |
|  |  |  |  |
| **INTRODUCTION** |  |  |  |
| Rationale | 3 | Describe the rationale for the review in the context of what is already known*, including mention of why a network meta-analysis has been conducted.* | ***3*** |
| Objectives | 4 | Provide an explicit statement of questions being addressed, with reference to participants, interventions, comparisons, outcomes, and study design (PICOS). | 4 |
|  |  |  |  |
| **METHODS** |  |  |  |
| Protocol and registration | 5 | Indicate whether a review protocol exists and if and where it can be accessed (e.g., Web address); and, if available, provide registration information, including registration number. | 5 |
| Eligibility criteria | 6 | Specify study characteristics (e.g., PICOS, length of follow-up) and report characteristics (e.g., years considered, language, publication status) used as criteria for eligibility, giving rationale. *Clearly describe eligible treatments included in the treatment network, and note whether any have been clustered or merged into the same node (with justification).* | ***5-6*** |
| Information sources | 7 | Describe all information sources (e.g., databases with dates of coverage, contact with study authors to identify additional studies) in the search and date last searched. | 5-6 |
| Search | 8 | Present full electronic search strategy for at least one database, including any limits used, such that it could be repeated. | 5-6 |
| Study selection | 9 | State the process for selecting studies (i.e., screening, eligibility, included in systematic review, and, if applicable, included in the meta-analysis). | 5-6 |
| Data collection process | 10 | Describe method of data extraction from reports (e.g., piloted forms, independently, in duplicate) and any processes for obtaining and confirming data from investigators. | 6 |
| Data items | 11 | List and define all variables for which data were sought (e.g., PICOS, funding sources) and any assumptions and simplifications made. | 6 |
| **Geometry of the network** | **S1** | Describe methods used to explore the geometry of the treatment network under study and potential biases related to it. This should include how the evidence base has been graphically summarized for presentation, and what characteristics were compiled and used to describe the evidence base to readers. | ***6*** |
| Risk of bias within individual studies | 12 | Describe methods used for assessing risk of bias of individual studies (including specification of whether this was done at the study or outcome level), and how this information is to be used in any data synthesis. | 7 |
| Summary measures | 13 | State the principal summary measures (e.g., risk ratio, difference in means). *Also describe the use of additional summary measures assessed, such as treatment rankings and surface under the cumulative ranking curve (SUCRA) values, as well as modified approaches used to present summary findings from meta-analyses.* | 7 |
| Planned methods of analysis | 14 | Describe the methods of handling data and combining results of studies for each network meta-analysis. This should include, but not be limited to:   - *Handling of multi-arm trials;* - *Selection of variance structure;* - *Selection of prior distributions in Bayesian analyses; and* - *Assessment of model fit.* | 5-7 |
| **Assessment of Inconsistency** | **S2** | Describe the statistical methods used to evaluate the agreement of direct and indirect evidence in the treatment network(s) studied. Describe efforts taken to address its presence when found. | 5-7 |
| Risk of bias across studies | 15 | Specify any assessment of risk of bias that may affect the cumulative evidence (e.g., publication bias, selective reporting within studies). | **5-7** |
| Additional analyses | 16 | Describe methods of additional analyses if done, indicating which were pre-specified. This may include, but not be limited to, the following:   - Sensitivity or subgroup analyses; - Meta-regression analyses; - *Alternative formulations of the treatment network; and* - *Use of alternative prior distributions for Bayesian analyses (if applicable).* | ***5-7*** |
|  |  |  |  |
| **RESULTS†** |  |  |  |
| Study selection | 17 | Give numbers of studies screened, assessed for eligibility, and included in the review, with reasons for exclusions at each stage, ideally with a flow diagram. | 8 |
| **Presentation of network structure** | **S3** | Provide a network graph of the included studies to enable visualization of the geometry of the treatment network. | *Appendix* |
| **Summary of network geometry** | **S4** | Provide a brief overview of characteristics of the treatment network. This may include commentary on the abundance of trials and randomized patients for the different interventions and pairwise comparisons in the network, gaps of evidence in the treatment network, and potential biases reflected by the network structure. | ***Appendix*** |
| Study characteristics | 18 | For each study, present characteristics for which data were extracted (e.g., study size, PICOS, follow-up period) and provide the citations. | Appendix |
| Risk of bias within studies | 19 | Present data on risk of bias of each study and, if available, any outcome level assessment. | Appendix |
| Results of individual studies | 20 | For all outcomes considered (benefits or harms), present, for each study: 1) simple summary data for each intervention group, and 2) effect estimates and confidence intervals. *Modified approaches may be needed to deal with information from larger networks.* | Appendix |
| Synthesis of results | 21 | Present results of each meta-analysis done, including confidence/credible intervals. *In larger networks, authors may focus on comparisons versus a particular comparator (e.g. placebo or standard care), with full findings presented in an appendix. League tables and forest plots may be considered to summarize pairwise comparisons.* If additional summary measures were explored (such as treatment rankings), these should also be presented. | Appendix |
| **Exploration for inconsistency** | **S5** | Describe results from investigations of inconsistency. This may include such information as measures of model fit to compare consistency and inconsistency models, *P* values from statistical tests, or summary of inconsistency estimates from different parts of the treatment network. | Appendix |
| Risk of bias across studies | 22 | Present results of any assessment of risk of bias across studies for the evidence base being studied. | Appendix |
| Results of additional analyses | 23 | Give results of additional analyses, if done (e.g., sensitivity or subgroup analyses, meta-regression analyses*, alternative network geometries studied, alternative choice of prior distributions for Bayesian analyses,* and so forth). | ***8-12*** |
|  |  |  |  |
| **DISCUSSION** |  |  |  |
| Summary of evidence | 24 | Summarize the main findings, including the strength of evidence for each main outcome; consider their relevance to key groups (e.g., healthcare providers, users, and policy-makers). | 12-17 |
| Limitations | 25 | Discuss limitations at study and outcome level (e.g., risk of bias), and at review level (e.g., incomplete retrieval of identified research, reporting bias). *Comment on the validity of the assumptions, such as transitivity and consistency. Comment on any concerns regarding network geometry (e.g., avoidance of certain comparisons).* | 16-17 |
| Conclusions | 26 | Provide a general interpretation of the results in the context of other evidence, and implications for future research. | 17 |
|  |  |  |  |
| **FUNDING** |  |  | Title |
| Funding | 27 | Describe sources of funding for the systematic review and other support (e.g., supply of data); role of funders for the systematic review. This should also include information regarding whether funding has been received from manufacturers of treatments in the network and/or whether some of the authors are content experts with professional conflicts of interest that could affect use of treatments in the network. |  |

PICOS = population, intervention, comparators, outcomes, study design.

* Text in italics indicateS wording specific to reporting of network meta-analyses that has been added to guidance from the PRISMA statement.

† Authors may wish to plan for use of appendices to present all relevant information in full detail for items in this section.

**Supplementary Figure 2.1** Preferred Reporting Items for Systematic Reviews and Meta-Analysis (PRISMA) flowchart of our analysis.


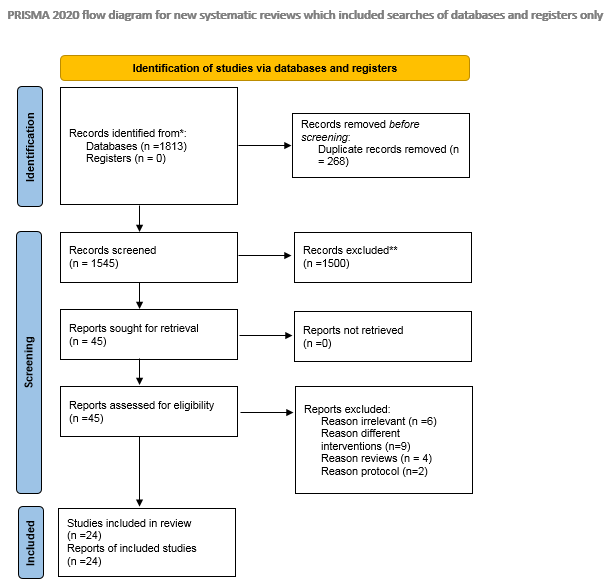


**Supplementary Figure 3.** Individual study results (for all studies) grouped by treatment comparison for 30-day mortality. RR: risk ratio.

**
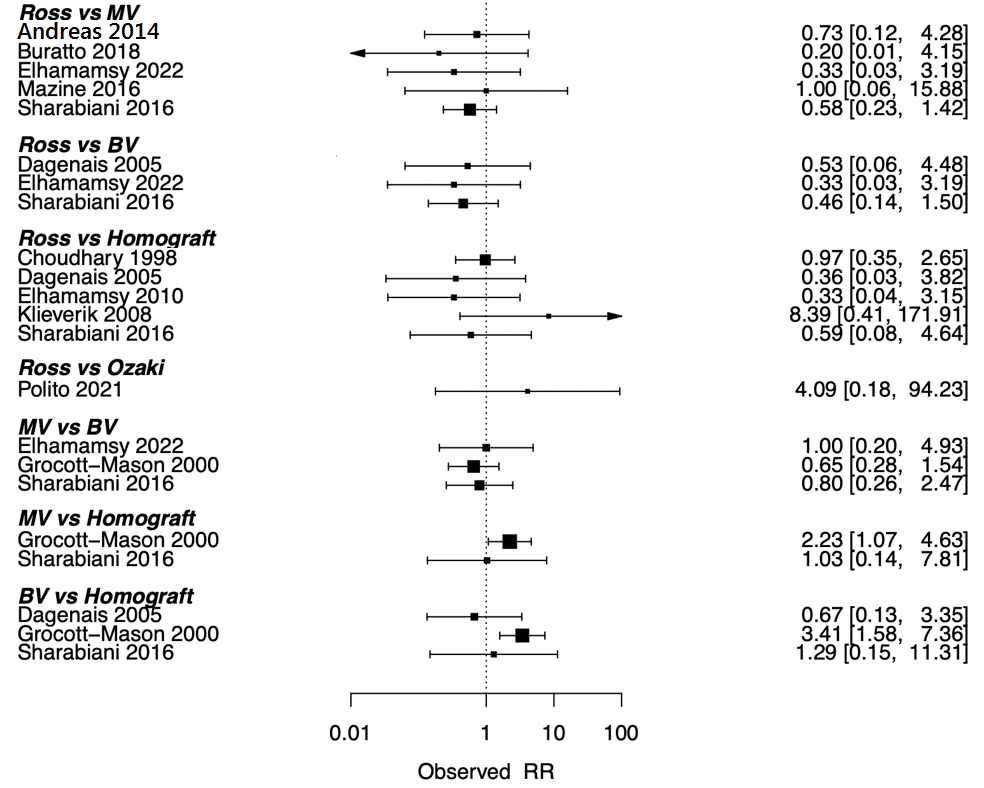
**

**Supplementary Figure 3.1.** Individual study results (for all studies) grouped by treatment comparison for 30-day stroke. RR: risk ratio.


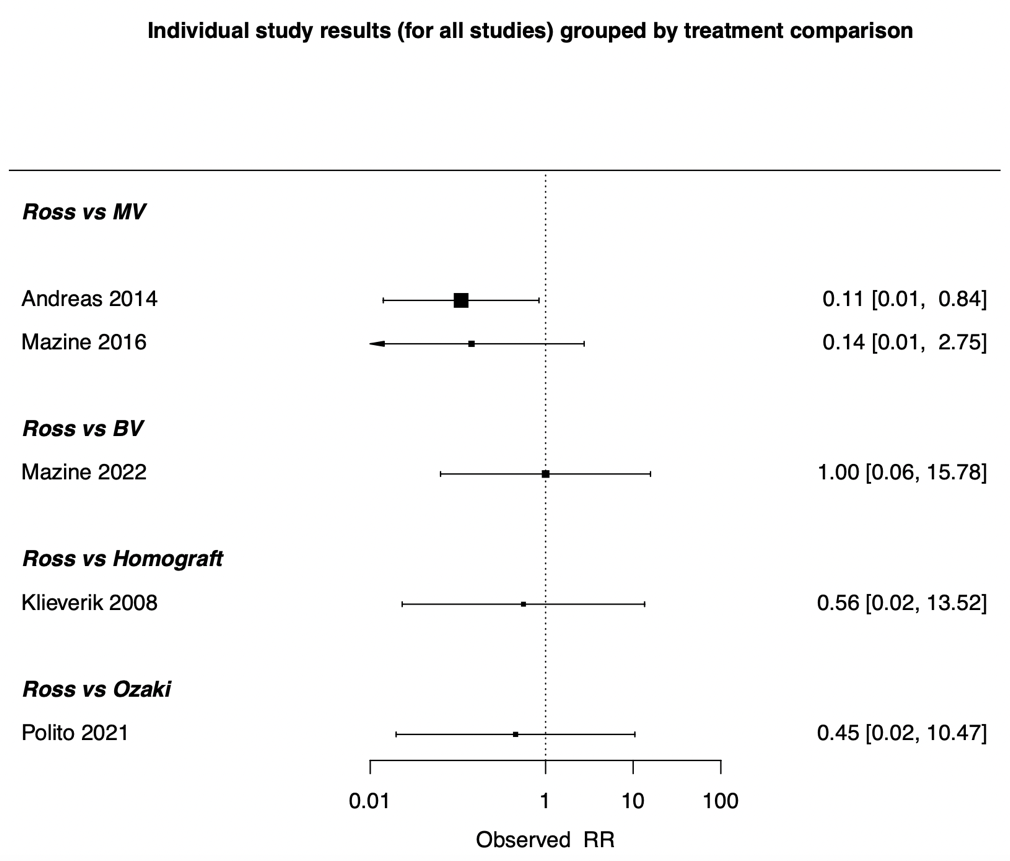


**Supplementary Figure 3.2.** Individual study results (for all studies) grouped by treatment comparison for 30-day myocardial infarction. RR: risk ratio.


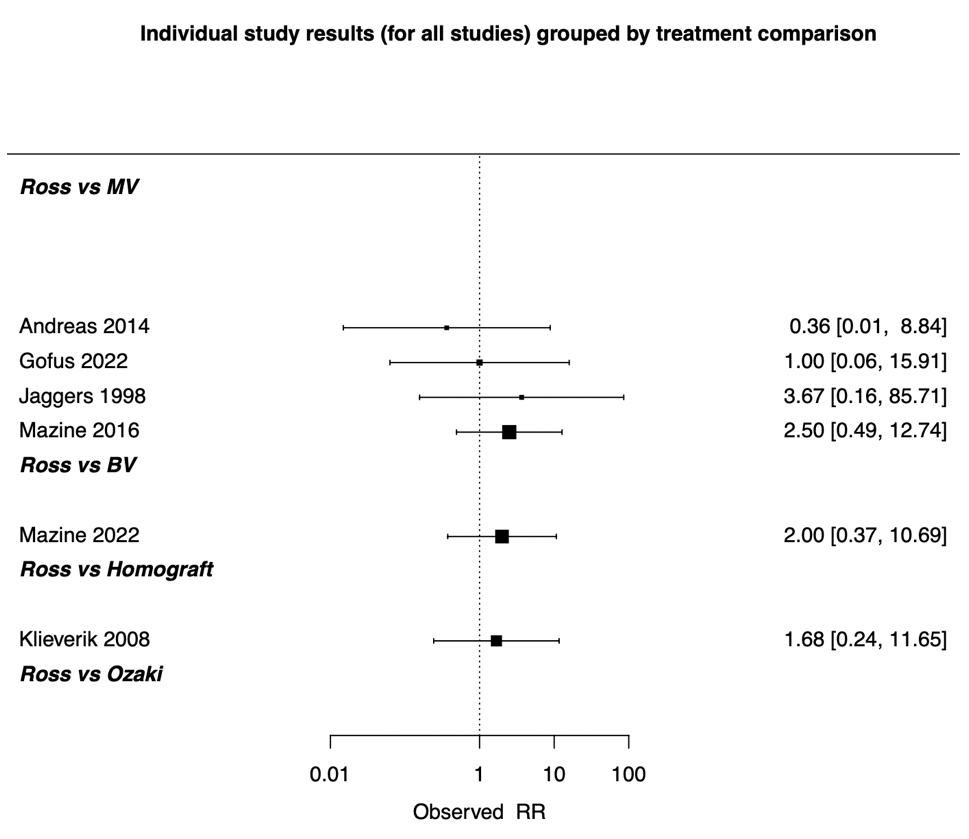


**Supplementary Figure 3.3.** Individual study results (for all studies) grouped by treatment comparison for 30-day major bleeding. RR: risk ratio.


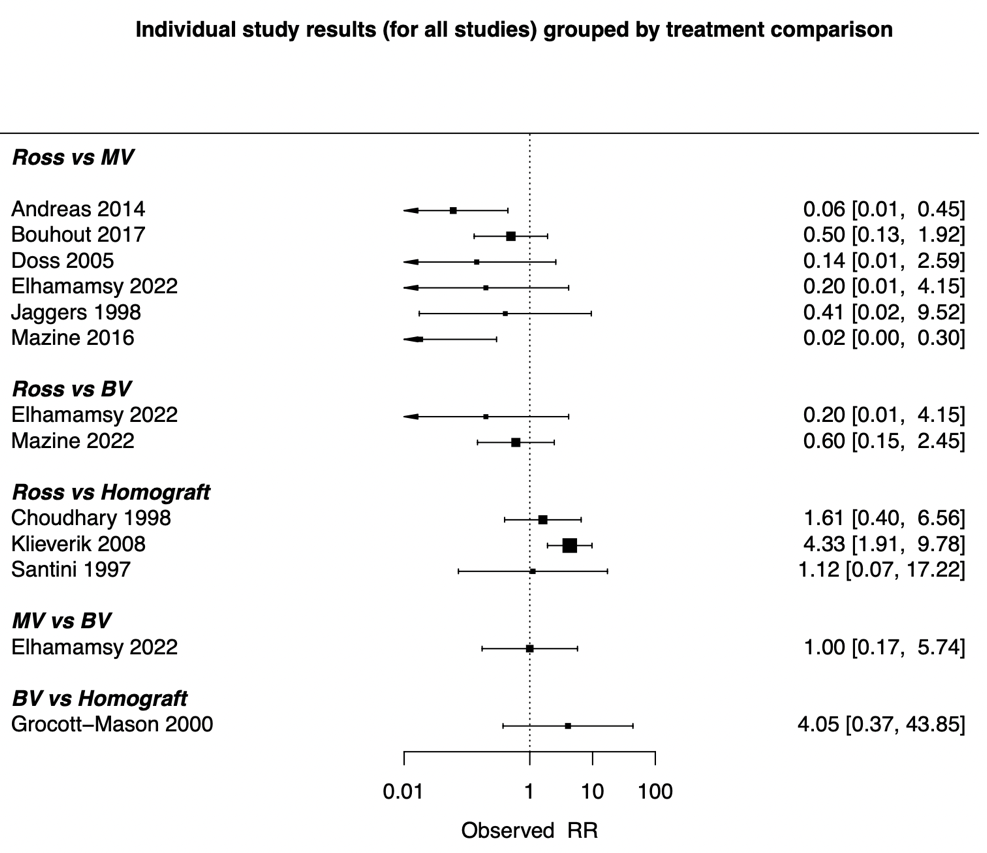


**Supplementary Figure 3.4.** Individual study results (for all studies) grouped by treatment comparison for long-term mortality. RR: risk ratio.


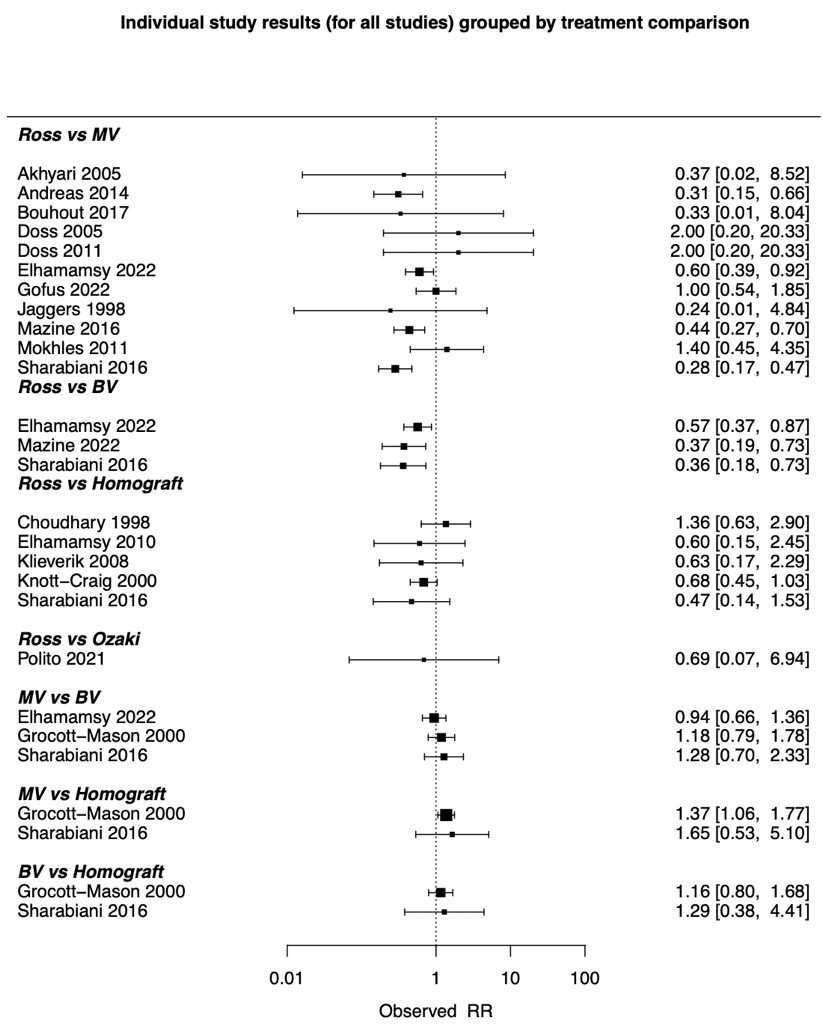


**Supplementary Figure 3.5.** Individual study results (for all studies) grouped by treatment comparison for long-term stroke. RR: risk ratio.


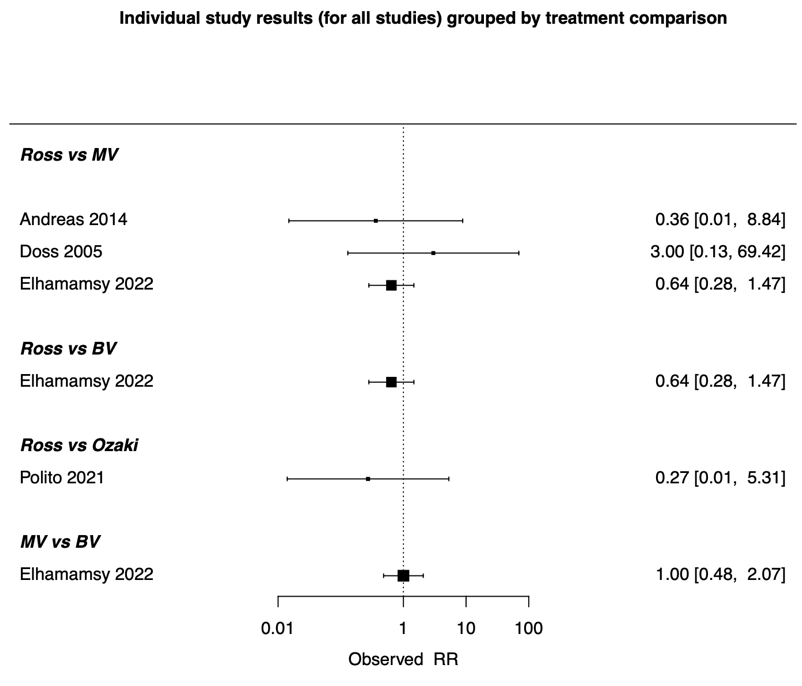


**Supplementary Figure 3.6.** Individual study results (for all studies) grouped by treatment comparison for long-term reintervention. RR: risk ratio.


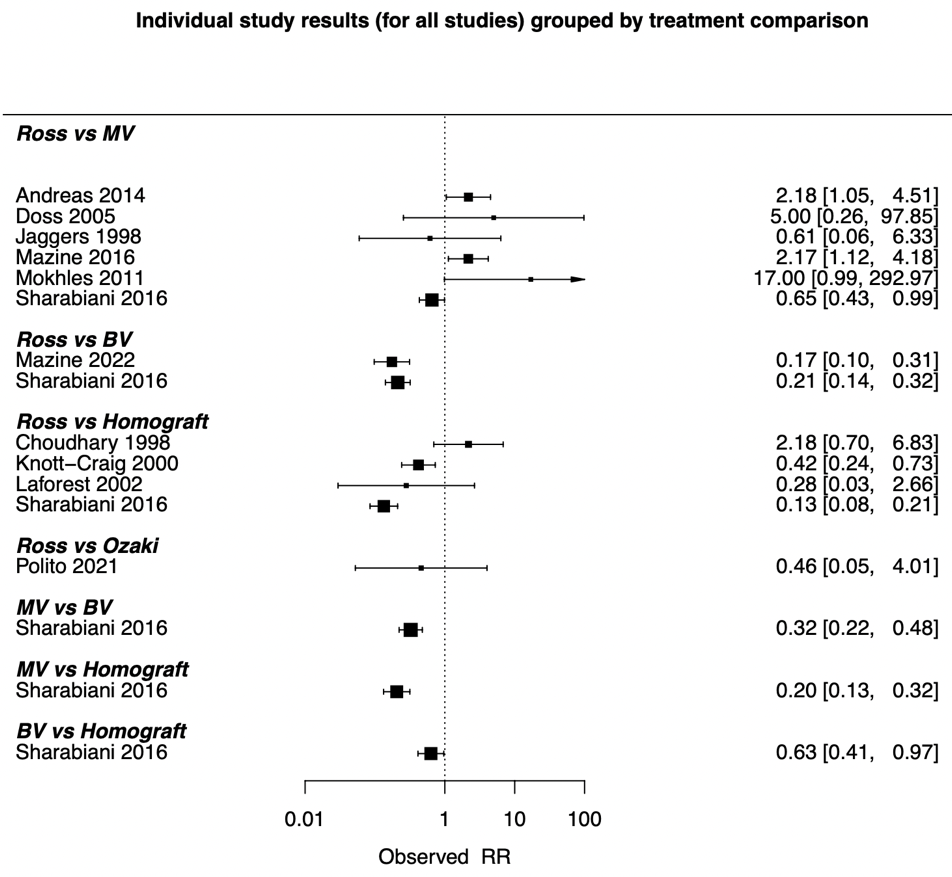


**Supplementary Figure 4.** Network plot of all studies for 30-day mortality. RR: risk ratio.

**
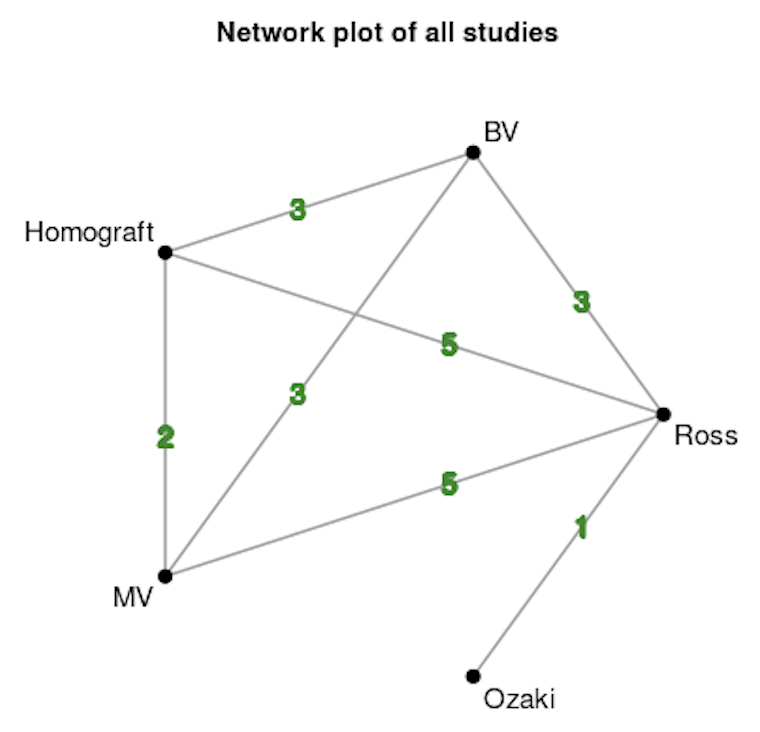
**

**Supplementary Figure 4.1.** Network plot of all studies for 30-day stroke. RR: risk ratio.

**
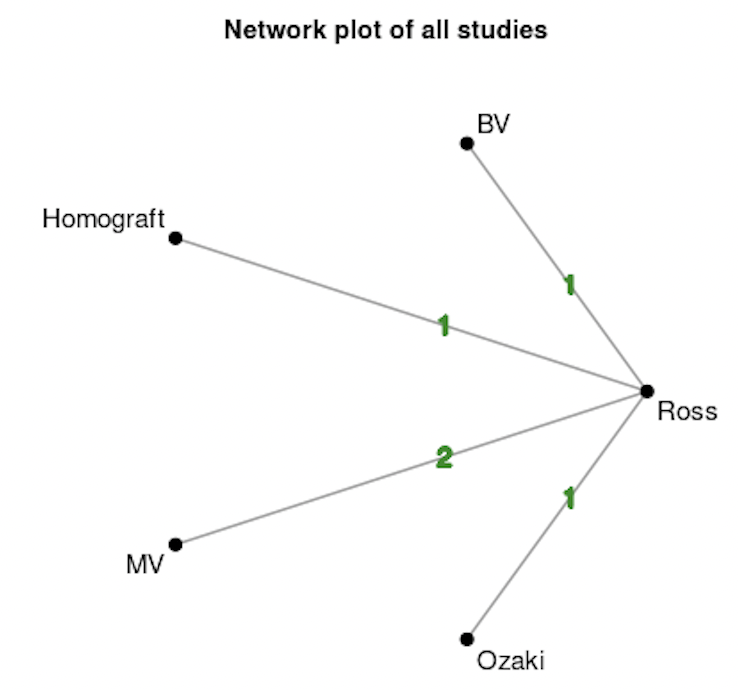
**

**Supplementary Figure 4.2.** Network plot of all studies for 30-day myocardial infarction. RR: risk ratio.


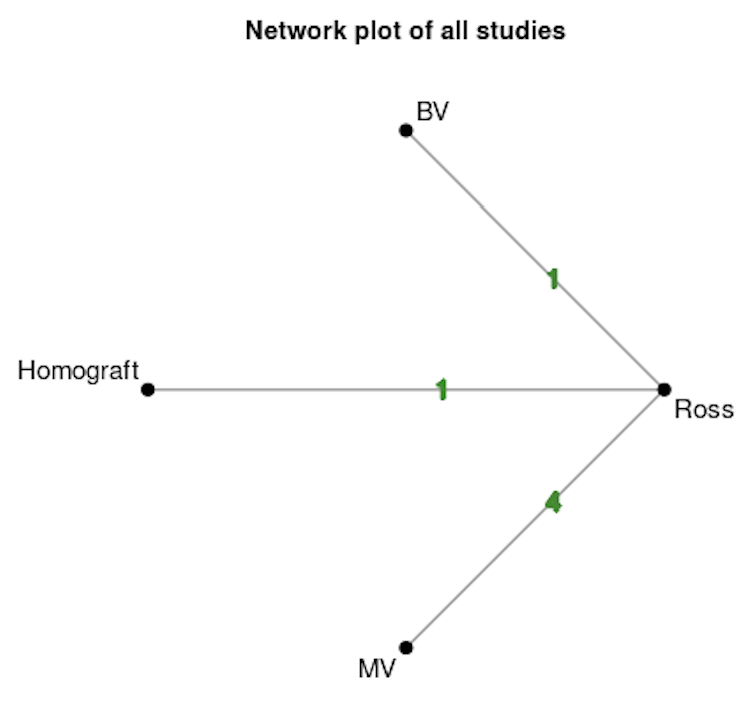


**Supplementary Figure 4.3.** Network plot of all studies for 30-day major bleeding. RR: risk ratio.


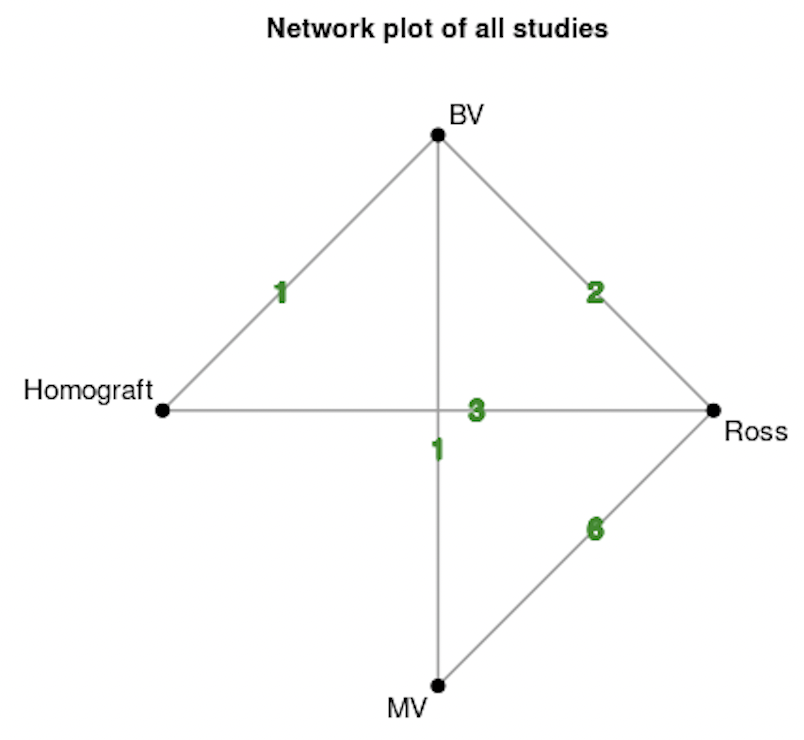


**Supplementary Figure 4.4.** Network plot of all studies for long-term mortality. RR: risk ratio.


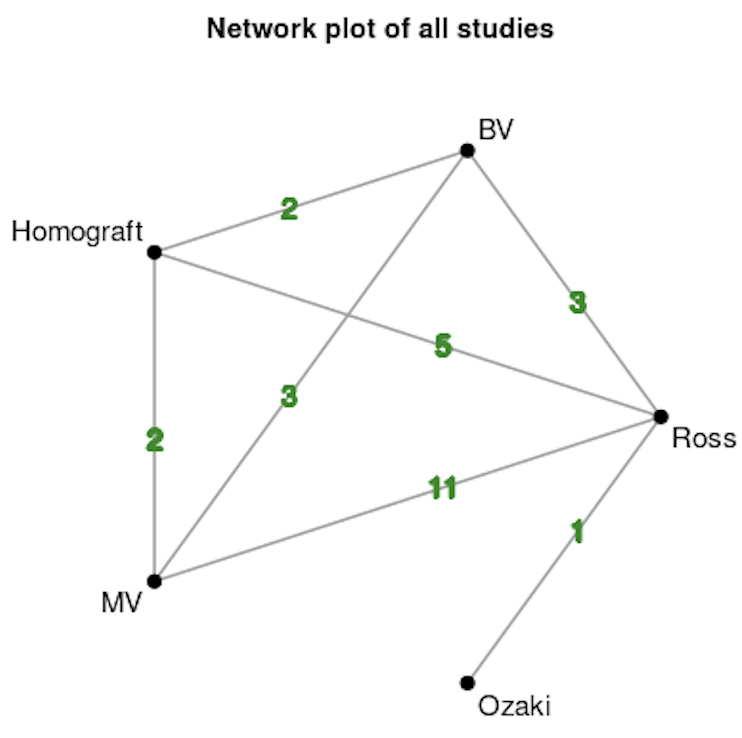


**Supplementary Figure 4.5.** Network plot of all studies for long-term stroke. RR: risk ratio.


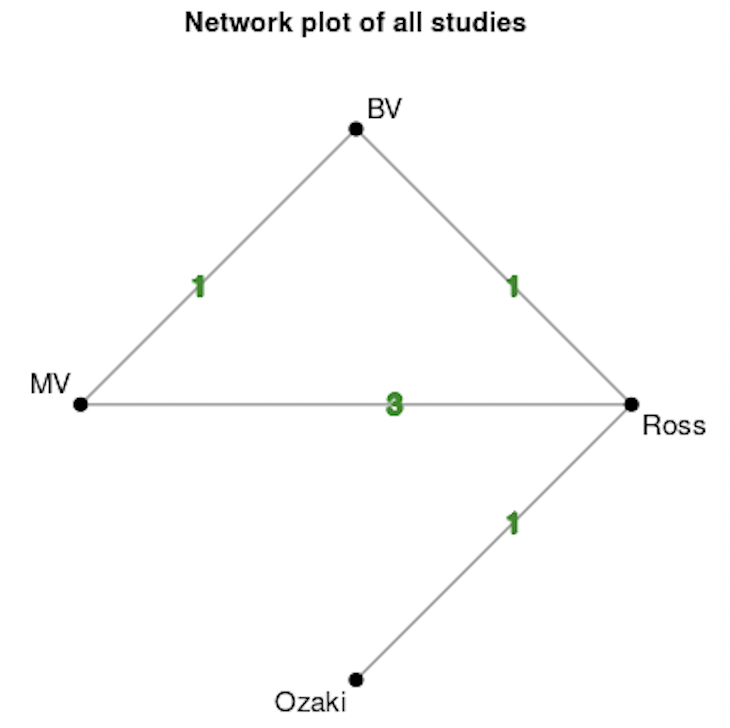


**Supplementary Figure 4.6.** Network plot of all studies for long-term reintervention. RR: risk ratio.


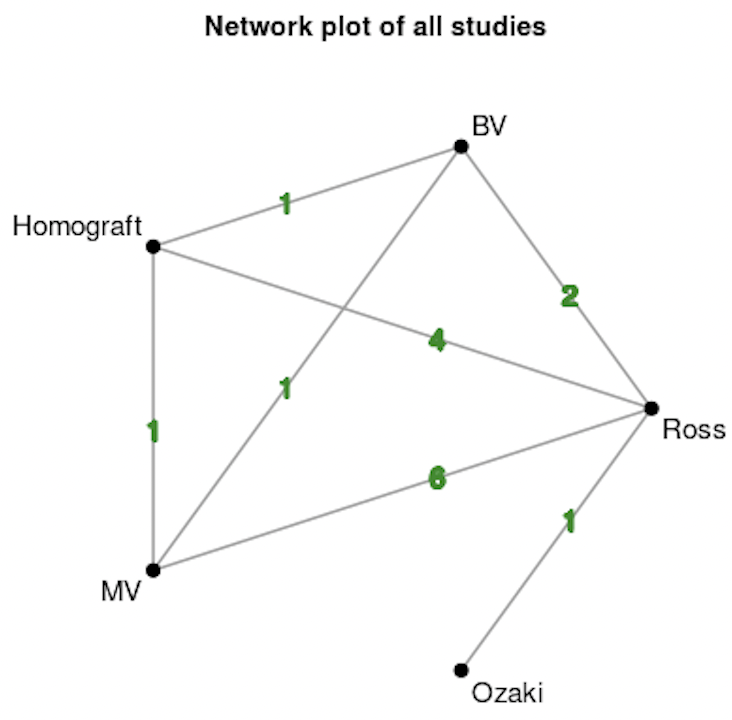


**Supplementary Table 1. Search strategy used and applied on different databases**

| Database | Search terms | Search field | Search results |
| --- | --- | --- | --- |
| PubMed | ((aortic stenosis OR Aortic Valve Stenoses) AND (mechanical valves OR prosthetic valve OR bioprostheses valves OR Bioprosthetic Valves OR biological valve OR tissue valve OR homograft OR ozaki procedure) AND (Ross procedure OR pulmonary valve transplantation OR pulmonary valve xenotransplantation OR pulmonary valve autograft OR autograft valve)) | All Fields | 813 |
| Cochrane | ((aortic stenosis OR Aortic Valve Stenoses) AND (mechanical valves OR prosthetic valve OR bioprostheses valves OR Bioprosthetic Valves OR biological valve OR tissue valve OR homograft OR ozaki procedure) AND (Ross procedure OR pulmonary valve transplantation OR pulmonary valve xenotransplantation OR pulmonary valve autograft OR autograft valve)) | All Fields | 258 |
| WOS | ((aortic stenosis OR Aortic Valve Stenoses) AND (mechanical valves OR prosthetic valve OR bioprostheses valves OR Bioprosthetic Valves OR biological valve OR tissue valve OR homograft OR ozaki procedure) AND (Ross procedure OR pulmonary valve transplantation OR pulmonary valve xenotransplantation OR pulmonary valve autograft OR autograft valve)) | All Field | 352 |
| Scopus | ((aortic stenosis OR Aortic Valve Stenoses) AND (mechanical valves OR prosthetic valve OR bioprostheses valves OR Bioprosthetic Valves OR biological valve OR tissue valve OR homograft OR ozaki procedure) AND (Ross procedure OR pulmonary valve transplantation OR pulmonary valve xenotransplantation OR pulmonary valve autograft OR autograft valve)) | Title and abstract | 170 |
| Embase | 1# Aortic stenosis  2# Aortic Valve Stenoses  3# Mechanical valves  4# Prosthetic valve  5# Bioprostheses valves  6# Bioprosthetic Valves  7# Biological valve  8# Tissue valve  9# Homograft  10# Ozaki procedure  11# Ross procedure  12# pulmonary valve transplantation 13# pulmonary valve  14# xenotransplantation pulmonary valve autograft  15# autograft valve  16# 1#OR2#  17# 3# OR 4# OR 5# OR 6# OR 7# OR 8# OR 9# OR 10#  18# 11# OR 12# OR 13# OR 14# OR 15#  19# 16# AND 17# AND 18# | Title and abstract | 220 |

**Supplementary Table 2.** Summary of the included studies.

| **Author** | **Study Type** | **Number of Patients** |
| --- | --- | --- |
| Akhyari/2005 | Retrospective Observational | 38 (18 Ross; 20 MV) |
| Andreas/2014 | Retrospective Observational | 332 (159 Ross; 173 MV) |
| Bouhout/2017 | Prospective Observational with PSM | 140 (70 Ross; 70 MV) |
| Buratto/2018 | Retrospective Observational with PSM | 550 (275 Ross; 275 MV) |
| Choudhary/1998 | Retrospective Observational | 189 (96 Ross; 93 Homograft) |
| Dagenais/2005 | Prospective Observational | 332 (76 Ross; 202 BV; 54 Homograft) |
| Doss/2005 | RCT | 40 (20 Ross; 20 MV) |
| Doss/2011 | Prospective Observational | 40 (20 Ross; 20 MV) |
| Elhamamsy/2010 | Prospective Observational | 216 (108 Ross; 108 Homograft) |
| Elhamamsy/2022 | Retrospective Observational with PSM | 1,302 (434 Ross; 434 MV; 434 BV) |
| Gofus/2022 | Retrospective Observational with PSM | 582 (291 Ross; 291 MV) |
| Grocott-Mason/2000 | Retrospective Observational | 518 (90 MV; 47 BV; 381 Homograft) |
| Heuvelman/2013 | Retrospective Observational | 40 (18 Ross; 9 MV; 13 Homograft) |
| Jaggers/1998 | Prospective Observational | 49 (22 Ross; 27 MV) |
| Klieverik/2008 | Prospective Observational | 169 (63 Ross; 106 Homograft) |
| Knott-Craig/2000 | Retrospective Observational | 238 (145 Ross; 93 Homograft) |
| Laforest/2002 | Prospective Observational | 243 (132 Ross; 111 Homograft) |
| Mazine/2016 | Retrospective Observational with PSM | 416 (208 Ross; 208 MV) |
| Mazine/2022 | Retrospective Observational with PSM | 216 (108 Ross; 108 BV) |
| Mokhles/2011 | Retrospective Observational with PSM | 506 (253 Ross; 253 MV) |
| Polito/2021/ | Retrospective Observational | 38 (16 Ross; 22 Ozaki) |
| Santini/1997 | RCT | 70 (33 Ross; 37 Homograft) |
| Sharabiani/2016 | Retrospective Observational with PSM | 1,501 (717 Ross; 567 MV; 164 BV; 53 Homograft) |
| Zacek/2016 | Retrospective Observational | 51 (22 Ross; 29 MV) |

**Supplementary Table 3.** Patient demographics, risk factors, and comorbidities of the included studies.

|  | **Age, mean (SD), median (IQR) / [Range]** | | | | | | | | | |
| --- | --- | --- | --- | --- | --- | --- | --- | --- | --- | --- |
|  | **Ross** | | **MV** | | **BV** | | **Homograft** | | **Ozaki** | |
| **Author/Year** | **Mean** | **SD** | **Mean** | **SD** | **Mean** | **SD** | **Mean** | **SD** | **Mean** | **SD** |
| Akhyari/2005 | 38 | 9.9 | 42.1 | 7.4 |  |  |  |  |  |  |
| Andreas/2014 | 35 | 8 | 41 | 7 |  |  |  |  |  |  |
| Bouhout/2017 | 52 / 13 |  | 52 / 14 |  |  |  |  |  |  |  |
| Buratto/2018 | 43 | 11 | 44 | 11 |  |  |  |  |  |  |
| Choudhary/1998 | 27.9 | 4.3 | 34.6 | 5.5 |  |  |  |  |  |  |
| Dagenais/2005 | 51.1 | 3.6 |  |  | 59.61 | 4.76 | 53.5 | 5.9 |  |  |
| Doss/2005 | 49 | 8.3 | 48 | 6.9 |  |  |  |  |  |  |
| Doss/2011 | 49 | 8.3 | 48 | 6.9 |  |  |  |  |  |  |
| Elhamamsy/2010 | 38 (19–66) |  |  |  |  |  | 39 | (19–68) |  |  |
| Elhamamsy/2022 | 35.9 | 9.2 | 36.7 | 8.8 | 36.2 | 9.4 |  |  |  |  |
| Gofus/2022 | 41.0 (34.0–48.0) |  | 42.0 | (32.0–52.0) |  |  |  |  |  |  |
| Grocott-Mason/2000 |  |  | 62.4 | 9.7 | 70.2 | 9 | 53.8 | 15.1 |  |  |
| Heuvelman/2013 |  |  |  |  |  |  |  |  |  |  |
| Jaggers/1998 | 38 | 10.8 | 41 | 11.3 |  |  |  |  |  |  |
| Klieverik/2008 | 29, 16-52 | 9 |  |  |  |  | 38, 16-55 | 10 |  |  |
| Knott-Craig/2000 | 35 | 13 |  |  |  |  | 49 | 17 |  |  |
| Laforest/2002 | 40 | 11 |  |  |  |  | 50 | 15 |  |  |
| Mazine/2016 | 37.3 | 9.5 | 37.1 | 10.9 |  |  |  |  |  |  |
| Mazine/2022 | 40 | (33-47) |  |  | 41 | (34-47) |  |  |  |  |
| Mokhles/2011 | 47.3 | 8.5 | 48.0 | 11.0 |  |  |  |  |  |  |
| Polito/2021/ | 11.1 / 6.6-14 |  |  |  |  |  |  |  | 13.9 / 9.8-16.2 |  |
| Santini/1997 | 29 | 15 |  |  |  |  | 39 | 15 |  |  |
| Sharabiani/2016 | 13.1 | (7.5–17.0) | 26.3 | (17.6–33.6) | 24.8 | (20.1–31.0) | 16.4 | (12.0–27.6) |  |  |
| Zacek/2016 | 37.8 | 11.9 | 39.7 | 7.3 |  |  |  |  |  |  |

|  | **Male (%)** | | | | |
| --- | --- | --- | --- | --- | --- |
|  | **Ross** | **MV** | **BV** | **Homograft** | **Ozaki** |
| **Author/Year** |  |  |  |  |  |
| Akhyari/2005 | 72.2 | 75 |  |  |  |
| Andreas/2014 | 80 | 75 |  |  |  |
| Bouhout/2017 | 77 | 67 |  |  |  |
| Buratto/2018 | 71 | 73 |  |  |  |
| Choudhary/1998 | 79.16 | 67.74 |  |  |  |
| Dagenais/2005 | 56.6 |  | 60.91 | 63 |  |
| Doss/2005 | 60 | 55 |  |  |  |
| Doss/2011 | 60 | 55 |  |  |  |
| Elhamamsy/2010 | 85 |  |  | 82 |  |
| Elhamamsy/2022 |  |  |  |  |  |
| Gofus/2022 | 75.6 | 75.9 |  |  |  |
| Grocott-Mason/2000 |  | 65.5 | 70.2 | 63.2 |  |
| Heuvelman/2013 |  |  |  |  |  |
| Jaggers/1998 | 77 | 63 |  |  |  |
| Klieverik/2008 |  |  |  |  |  |
| Knott-Craig/2000 | 55 |  |  | 80 |  |
| Laforest/2002 | 62 |  |  | 66 |  |
| Mazine/2016 | 133 | 130 |  |  |  |
| Mazine/2022 | 64 |  | 74 |  |  |
| Mokhles/2011 | 76.3 | 73.1 |  |  |  |
| Polito/2021/ |  |  |  |  |  |
| Santini/1997 | 85 |  |  | 92 |  |
| Sharabiani/2016 | 71.6 | 78.7 | 63.8 | 51 |  |
| Zacek/2016 | 83 | 69 |  |  |  |

|  | **Euroscore 2 mean (SD), median / range** | | | | | | | | | |
| --- | --- | --- | --- | --- | --- | --- | --- | --- | --- | --- |
|  | **Ross** | | **MV** | | **BV** | | **Homograft** | | **Ozaki** | |
| **Author/Year** | **Mean** | **SD** | **Mean** | **SD** | **Mean** | **SD** | **Mean** | **SD** | **Mean** | **SD** |
| Akhyari/2005 |  |  |  |  |  |  |  |  |  |  |
| Andreas/2014 |  |  |  |  |  |  |  |  |  |  |
| Bouhout/2017 | 0.96 |  | 0.86 |  |  |  |  |  |  |  |
| Buratto/2018 |  |  |  |  |  |  |  |  |  |  |
| Choudhary/1998 |  |  |  |  |  |  |  |  |  |  |
| Dagenais/2005 |  |  |  |  |  |  |  |  |  |  |
| Doss/2005 |  |  |  |  |  |  |  |  |  |  |
| Doss/2011 |  |  |  |  |  |  |  |  |  |  |
| Elhamamsy/2010 |  |  |  |  |  |  |  |  |  |  |
| Elhamamsy/2022 |  |  |  |  |  |  |  |  |  |  |
| Gofus/2022 |  |  |  |  |  |  |  |  |  |  |
| Grocott-Mason/2000 |  |  |  |  |  |  |  |  |  |  |
| Heuvelman/2013 |  |  |  |  |  |  |  |  |  |  |
| Jaggers/1998 |  |  |  |  |  |  |  |  |  |  |
| Klieverik/2008 |  |  |  |  |  |  |  |  |  |  |
| Knott-Craig/2000 |  |  |  |  |  |  |  |  |  |  |
| Laforest/2002 |  |  |  |  |  |  |  |  |  |  |
| Mazine/2016 |  |  |  |  |  |  |  |  |  |  |
| Mazine/2022 |  |  |  |  |  |  |  |  |  |  |
| Mokhles/2011 |  |  |  |  |  |  |  |  |  |  |
| Polito/2021/ |  |  |  |  |  |  |  |  |  |  |
| Santini/1997 |  |  |  |  |  |  |  |  |  |  |
| Sharabiani/2016 |  |  |  |  |  |  |  |  |  |  |
| Zacek/2016 |  |  |  |  |  |  |  |  |  |  |

|  | **NYHA Functional Class III/IV (%)** | | | | |
| --- | --- | --- | --- | --- | --- |
|  | **Ross** | **MV** | **BV** | **Homograft** | **Ozaki** |
| **Author/Year** |  |  |  |  |  |
| Akhyari/2005 |  |  |  |  |  |
| Andreas/2014 | 45 | 55 |  |  |  |
| Bouhout/2017 | 44 | 42 |  |  |  |
| Buratto/2018 | 17 | 16 |  |  |  |
| Choudhary/1998 |  |  |  |  |  |
| Dagenais/2005 |  |  |  |  |  |
| Doss/2005 | 16 | 16 |  |  |  |
| Doss/2011 | 80 | 80 |  |  |  |
| Elhamamsy/2010 | 24 |  |  | 35 |  |
| Elhamamsy/2022 |  |  |  |  |  |
| Gofus/2022 | 9.9 | 0.7 |  |  |  |
| Grocott-Mason/2000 |  | 100 | 100 | 100 |  |
| Heuvelman/2013 |  |  |  |  |  |
| Jaggers/1998 |  |  |  |  |  |
| Klieverik/2008 | 20 |  |  | 30 |  |
| Knott-Craig/2000 |  |  |  |  |  |
| Laforest/2002 | 43 |  |  | 57 |  |
| Mazine/2016 | 38 | 66 |  |  |  |
| Mazine/2022 | 16 |  | 16 |  |  |
| Mokhles/2011 | 35.6 | 37.5 |  |  |  |
| Polito/2021/ |  |  |  |  |  |
| Santini/1997 | 21 |  |  | 21 |  |
| Sharabiani/2016 |  |  |  |  |  |
| Zacek/2016 | 13 | 14 |  |  |  |

|  | **BSA (kg), mean (SD), median / range** | | | | | | | | | |
| --- | --- | --- | --- | --- | --- | --- | --- | --- | --- | --- |
|  | **Ross** | | **MV** | | **BV** | | **Homograft** | | **Ozaki** | |
| **Author/Year** | **Mean** | **SD** | **Mean** | **SD** | **Mean** | **SD** | **Mean** | **SD** | **Mean** | **SD** |
| Akhyari/2005 |  |  |  |  |  |  |  |  |  |  |
| Andreas/2014 |  |  |  |  |  |  |  |  |  |  |
| Bouhout/2017 |  |  |  |  |  |  |  |  |  |  |
| Buratto/2018 |  |  |  |  |  |  |  |  |  |  |
| Choudhary/1998 |  |  |  |  |  |  |  |  |  |  |
| Dagenais/2005 |  |  |  |  |  |  |  |  |  |  |
| Doss/2005 | 1.82 | 0.7 | 1.89 | 0.4 |  |  |  |  |  |  |
| Doss/2011 | 1.82 | 0.7 | 1.89 | 0.4 |  |  |  |  |  |  |
| Elhamamsy/2010 | 1.9 | 0.2 |  |  |  |  | 1.9 | 0.2 |  |  |
| Elhamamsy/2022 |  |  |  |  |  |  |  |  |  |  |
| Gofus/2022 |  |  |  |  |  |  |  |  |  |  |
| Grocott-Mason/2000 |  |  |  |  |  |  |  |  |  |  |
| Heuvelman/2013 |  |  |  |  |  |  |  |  |  |  |
| Jaggers/1998 |  |  |  |  |  |  |  |  |  |  |
| Klieverik/2008 |  |  |  |  |  |  |  |  |  |  |
| Knott-Craig/2000 |  |  |  |  |  |  |  |  |  |  |
| Laforest/2002 | 1.79 | 0.23 |  |  |  |  | 1.78 | 0.22 |  |  |
| Mazine/2016 |  |  |  |  |  |  |  |  |  |  |
| Mazine/2022 | 1.9 | (1.7-2.0) |  |  | 1.9 | (1.8-2.1) |  |  |  |  |
| Mokhles/2011 |  |  |  |  |  |  |  |  |  |  |
| Polito/2021/ |  |  |  |  |  |  |  |  |  |  |
| Santini/1997 |  |  |  |  |  |  |  |  |  |  |
| Sharabiani/2016 |  |  |  |  |  |  |  |  |  |  |
| Zacek/2016 |  |  |  |  |  |  |  |  |  |  |

|  | **BMI (kg/m^2), mean (SD), median / range** | | | | | | | | | |
| --- | --- | --- | --- | --- | --- | --- | --- | --- | --- | --- |
|  | **Ross** | | **MV** | | **BV** | | **Homograft** | | **Ozaki** | |
| **Author/Year** | **Mean** | **SD** | **Mean** | **SD** | **Mean** | **SD** | **Mean** | **SD** | **Mean** | **SD** |
| Akhyari/2005 |  |  |  |  |  |  |  |  |  |  |
| Andreas/2014 | 23.9 | 3.2 | 26.9 | 7.0 |  |  |  |  |  |  |
| Bouhout/2017 |  |  |  |  |  |  |  |  |  |  |
| Buratto/2018 |  |  |  |  |  |  |  |  |  |  |
| Choudhary/1998 |  |  |  |  |  |  |  |  |  |  |
| Dagenais/2005 |  |  |  |  |  |  |  |  |  |  |
| Doss/2005 |  |  |  |  |  |  |  |  |  |  |
| Doss/2011 |  |  |  |  |  |  |  |  |  |  |
| Elhamamsy/2010 |  |  |  |  |  |  |  |  |  |  |
| Elhamamsy/2022 |  |  |  |  |  |  |  |  |  |  |
| Gofus/2022 | 26.7 | (23.9–29.4) | 26.3 | (23.6–29.9) |  |  |  |  |  |  |
| Grocott-Mason/2000 |  |  |  |  |  |  |  |  |  |  |
| Heuvelman/2013 |  |  |  |  |  |  |  |  |  |  |
| Jaggers/1998 |  |  |  |  |  |  |  |  |  |  |
| Klieverik/2008 |  |  |  |  |  |  |  |  |  |  |
| Knott-Craig/2000 |  |  |  |  |  |  |  |  |  |  |
| Laforest/2002 |  |  |  |  |  |  |  |  |  |  |
| Mazine/2016 |  |  |  |  |  |  |  |  |  |  |
| Mazine/2022 |  |  |  |  |  |  |  |  |  |  |
| Mokhles/2011 |  |  |  |  |  |  |  |  |  |  |
| Polito/2021/ |  |  |  |  |  |  |  |  |  |  |
| Santini/1997 |  |  |  |  |  |  |  |  |  |  |
| Sharabiani/2016 |  |  |  |  |  |  |  |  |  |  |
| Zacek/2016 | 23.9 | 3.2 | 26.9 | 7.0 |  |  |  |  |  |  |

|  | **Hypertension (%)** | | | | |
| --- | --- | --- | --- | --- | --- |
|  | **Ross** | **MV** | **BV** | **Homograft** | **Ozaki** |
| **Author/Year** |  |  |  |  |  |
| Akhyari/2005 | 11.11 | 20 |  |  |  |
| Andreas/2014 |  |  |  |  |  |
| Bouhout/2017 | 24 | 20 |  |  |  |
| Buratto/2018 | 21 | 21 |  |  |  |
| Choudhary/1998 |  |  |  |  |  |
| Dagenais/2005 | 27.6 |  | 40.58 | 40.7 |  |
| Doss/2005 | 35 | 30 |  |  |  |
| Doss/2011 | 35 | 30 |  |  |  |
| Elhamamsy/2010 |  |  |  |  |  |
| Elhamamsy/2022 | 18 | 19 | 18 |  |  |
| Gofus/2022 | 33.7 | 41.9 |  |  |  |
| Grocott-Mason/2000 |  | 27.8 | 17 | 10.2 |  |
| Heuvelman/2013 |  |  |  |  |  |
| Jaggers/1998 |  |  |  |  |  |
| Klieverik/2008 |  |  |  |  |  |
| Knott-Craig/2000 |  |  |  |  |  |
| Laforest/2002 |  |  |  |  |  |
| Mazine/2016 | 41 | 42 |  |  |  |
| Mazine/2022 | 18 |  | 18 |  |  |
| Mokhles/2011 | 31.6 | 34 |  |  |  |
| Polito/2021/ |  |  |  |  |  |
| Santini/1997 |  |  |  |  |  |
| Sharabiani/2016 |  |  |  |  |  |
| Zacek/2016 | 22 | 31 |  |  |  |

|  | **Hypercholesterolemia (%)** | | | | |
| --- | --- | --- | --- | --- | --- |
|  | **Ross** | **MV** | **BV** | **Homograft** | **Ozaki** |
| **Author/Year** |  |  |  |  |  |
| Akhyari/2005 | 5.56 | 20 |  |  |  |
| Andreas/2014 |  |  |  |  |  |
| Bouhout/2017 | 20 | 17 |  |  |  |
| Buratto/2018 |  |  |  |  |  |
| Choudhary/1998 |  |  |  |  |  |
| Dagenais/2005 |  |  |  |  |  |
| Doss/2005 |  |  |  |  |  |
| Doss/2011 | 19 |  |  | 27 |  |
| Elhamamsy/2010 |  |  |  |  |  |
| Elhamamsy/2022 |  |  |  |  |  |
| Gofus/2022 | 16.8 | 22 |  |  |  |
| Grocott-Mason/2000 |  |  |  |  |  |
| Heuvelman/2013 |  |  |  |  |  |
| Jaggers/1998 |  |  |  |  |  |
| Klieverik/2008 |  |  |  |  |  |
| Knott-Craig/2000 |  |  |  |  |  |
| Laforest/2002 |  |  |  |  |  |
| Mazine/2016 | 27 | 20 |  |  |  |
| Mazine/2022 | 16 |  | 10 |  |  |
| Mokhles/2011 |  |  |  |  |  |
| Polito/2021/ |  |  |  |  |  |
| Santini/1997 |  |  |  |  |  |
| Sharabiani/2016 |  |  |  |  |  |
| Zacek/2016 |  |  |  |  |  |

|  | **Prior Stroke/TIA (%)** | | | | |
| --- | --- | --- | --- | --- | --- |
|  | **Ross** | **MV** | **BV** | **Homograft** | **Ozaki** |
| **Author/Year** |  |  |  |  |  |
| Akhyari/2005 |  |  |  |  |  |
| Andreas/2014 |  |  |  |  |  |
| Bouhout/2017 |  |  |  |  |  |
| Buratto/2018 |  |  |  |  |  |
| Choudhary/1998 |  |  |  |  |  |
| Dagenais/2005 |  |  |  |  |  |
| Doss/2005 |  |  |  |  |  |
| Doss/2011 |  |  |  |  |  |
| Elhamamsy/2010 |  |  |  |  |  |
| Elhamamsy/2022 |  |  |  |  |  |
| Gofus/2022 |  |  |  |  |  |
| Grocott-Mason/2000 |  |  |  |  |  |
| Heuvelman/2013 |  |  |  |  |  |
| Jaggers/1998 |  |  |  |  |  |
| Klieverik/2008 |  |  |  |  |  |
| Knott-Craig/2000 |  |  |  |  |  |
| Laforest/2002 |  |  |  |  |  |
| Mazine/2016 |  |  |  |  |  |
| Mazine/2022 | 3 |  | 1 |  |  |
| Mokhles/2011 |  |  |  |  |  |
| Polito/2021/ |  |  |  |  |  |
| Santini/1997 |  |  |  |  |  |
| Sharabiani/2016 |  |  |  |  |  |
| Zacek/2016 |  |  |  |  |  |

|  | **Previous Stroke (%)** | | | | |
| --- | --- | --- | --- | --- | --- |
|  | **Ross** | **MV** | **BV** | **Homograft** | **Ozaki** |
| **Author/Year** |  |  |  |  |  |
| Akhyari/2005 |  |  |  |  |  |
| Andreas/2014 |  |  |  |  |  |
| Bouhout/2017 |  |  |  |  |  |
| Buratto/2018 |  |  |  |  |  |
| Choudhary/1998 |  |  |  |  |  |
| Dagenais/2005 |  |  |  |  |  |
| Doss/2005 |  |  |  |  |  |
| Doss/2011 |  |  |  |  |  |
| Elhamamsy/2010 |  |  |  |  |  |
| Elhamamsy/2022 |  |  |  |  |  |
| Gofus/2022 |  |  |  |  |  |
| Grocott-Mason/2000 |  |  |  |  |  |
| Heuvelman/2013 |  |  |  |  |  |
| Jaggers/1998 |  |  |  |  |  |
| Klieverik/2008 |  |  |  |  |  |
| Knott-Craig/2000 |  |  |  |  |  |
| Laforest/2002 |  |  |  |  |  |
| Mazine/2016 | 7 | 10 |  |  |  |
| Mazine/2022 |  |  |  |  |  |
| Mokhles/2011 |  |  |  |  |  |
| Polito/2021/ |  |  |  |  |  |
| Santini/1997 |  |  |  |  |  |
| Sharabiani/2016 |  |  |  |  |  |
| Zacek/2016 |  |  |  |  |  |

|  | **Atrial Fibrillation (%)** | | | | |
| --- | --- | --- | --- | --- | --- |
|  | **Ross** | **MV** | **BV** | **Homograft** | **Ozaki** |
| **Author/Year** |  |  |  |  |  |
| Akhyari/2005 |  |  |  |  |  |
| Andreas/2014 |  |  |  |  |  |
| Bouhout/2017 | 1 | 3 |  |  |  |
| Buratto/2018 |  |  |  |  |  |
| Choudhary/1998 |  |  |  |  |  |
| Dagenais/2005 | 1.3 |  | 3.95 | 7.4 |  |
| Doss/2005 | 0 | 5 |  |  |  |
| Doss/2011 | 0 | 5 |  |  |  |
| Elhamamsy/2010 | 1 |  |  | 5 |  |
| Elhamamsy/2022 | 4 | 3 | 4 |  |  |
| Gofus/2022 |  |  |  |  |  |
| Grocott-Mason/2000 |  | 6.7 | 17 | 5.2 |  |
| Heuvelman/2013 |  |  |  |  |  |
| Jaggers/1998 |  |  |  |  |  |
| Klieverik/2008 |  |  |  |  |  |
| Knott-Craig/2000 |  |  |  |  |  |
| Laforest/2002 |  |  |  |  |  |
| Mazine/2016 | 2 | 7 |  |  |  |
| Mazine/2022 | 1 |  | 1 |  |  |
| Mokhles/2011 |  |  |  |  |  |
| Polito/2021/ |  |  |  |  |  |
| Santini/1997 |  |  |  |  |  |
| Sharabiani/2016 |  |  |  |  |  |
| Zacek/2016 |  |  |  |  |  |

|  | **Smoking/Tobacco Use (%)** | | | | |
| --- | --- | --- | --- | --- | --- |
|  | **Ross** | **MV** | **BV** | **Homograft** | **Ozaki** |
| **Author/Year** |  |  |  |  |  |
| Akhyari/2005 | 5.56 | 15 |  |  |  |
| Andreas/2014 |  |  |  |  |  |
| Bouhout/2017 | 27 | 31 |  |  |  |
| Buratto/2018 |  |  |  |  |  |
| Choudhary/1998 |  |  |  |  |  |
| Dagenais/2005 | 24 |  | 16.24 | 17.3 |  |
| Doss/2005 |  |  |  |  |  |
| Doss/2011 |  |  |  |  |  |
| Elhamamsy/2010 | 17 |  |  | 21 |  |
| Elhamamsy/2022 |  |  |  |  |  |
| Gofus/2022 | 20.3 | 21 |  |  |  |
| Grocott-Mason/2000 |  |  |  |  |  |
| Heuvelman/2013 |  |  |  |  |  |
| Jaggers/1998 |  |  |  |  |  |
| Klieverik/2008 |  |  |  |  |  |
| Knott-Craig/2000 |  |  |  |  |  |
| Laforest/2002 |  |  |  |  |  |
| Mazine/2016 | 102 | 80 |  |  |  |
| Mazine/2022 | 45 |  | 45 |  |  |
| Mokhles/2011 |  |  |  |  |  |
| Polito/2021/ |  |  |  |  |  |
| Santini/1997 |  |  |  |  |  |
| Sharabiani/2016 |  |  |  |  |  |
| Zacek/2016 |  |  |  |  |  |

|  | **Peripheral Vascular Disease (%)** | | | | |
| --- | --- | --- | --- | --- | --- |
|  | **Ross** | **MV** | **BV** | **Homograft** | **Ozaki** |
| **Author/Year** |  |  |  |  |  |
| Akhyari/2005 |  |  |  |  |  |
| Andreas/2014 |  |  |  |  |  |
| Bouhout/2017 |  |  |  |  |  |
| Buratto/2018 | 0 | 0 |  |  |  |
| Choudhary/1998 |  |  |  |  |  |
| Dagenais/2005 | 6.6 |  | 5.5 | 16.7 |  |
| Doss/2005 |  |  |  |  |  |
| Doss/2011 |  |  |  |  |  |
| Elhamamsy/2010 |  |  |  |  |  |
| Elhamamsy/2022 |  |  |  |  |  |
| Gofus/2022 |  |  |  |  |  |
| Grocott-Mason/2000 |  |  |  |  |  |
| Heuvelman/2013 |  |  |  |  |  |
| Jaggers/1998 |  |  |  |  |  |
| Klieverik/2008 |  |  |  |  |  |
| Knott-Craig/2000 |  |  |  |  |  |
| Laforest/2002 |  |  |  |  |  |
| Mazine/2016 |  |  |  |  |  |
| Mazine/2022 |  |  |  |  |  |
| Mokhles/2011 |  |  |  |  |  |
| Polito/2021/ |  |  |  |  |  |
| Santini/1997 |  |  |  |  |  |
| Sharabiani/2016 |  |  |  |  |  |
| Zacek/2016 |  |  |  |  |  |

|  | **Chronic Obstructive Pulmonary Disease (%)** | | | | |
| --- | --- | --- | --- | --- | --- |
|  | **Ross** | **MV** | **BV** | **Homograft** | **Ozaki** |
| **Author/Year** |  |  |  |  |  |
| Akhyari/2005 |  |  |  |  |  |
| Andreas/2014 |  |  |  |  |  |
| Bouhout/2017 | 8 | 4 |  |  |  |
| Buratto/2018 | 4 | 5 |  |  |  |
| Choudhary/1998 |  |  |  |  |  |
| Dagenais/2005 | 7.9 |  | 17.28 | 9.4 |  |
| Doss/2005 |  |  |  |  |  |
| Doss/2011 |  |  |  |  |  |
| Elhamamsy/2010 |  |  |  |  |  |
| Elhamamsy/2022 | 5 | 3 | 4 |  |  |
| Gofus/2022 | 6.2 | 6.5 |  |  |  |
| Grocott-Mason/2000 |  |  |  |  |  |
| Heuvelman/2013 |  |  |  |  |  |
| Jaggers/1998 |  |  |  |  |  |
| Klieverik/2008 |  |  |  |  |  |
| Knott-Craig/2000 |  |  |  |  |  |
| Laforest/2002 |  |  |  |  |  |
| Mazine/2016 | 4 | 1 |  |  |  |
| Mazine/2022 | 1 |  | 1 |  |  |
| Mokhles/2011 |  |  |  |  |  |
| Polito/2021/ |  |  |  |  |  |
| Santini/1997 |  |  |  |  |  |
| Sharabiani/2016 |  |  |  |  |  |
| Zacek/2016 |  |  |  |  |  |

|  | **Renal Failure (%)** | | | | |
| --- | --- | --- | --- | --- | --- |
|  | **Ross** | **MV** | **BV** | **Homograft** | **Ozaki** |
| **Author/Year** |  |  |  |  |  |
| Akhyari/2005 | 0 | 0 |  |  |  |
| Andreas/2014 |  |  |  |  |  |
| Bouhout/2017 | 3 | 7 |  |  |  |
| Buratto/2018 | 0 | 0 |  |  |  |
| Choudhary/1998 |  |  |  |  |  |
| Dagenais/2005 |  |  |  |  |  |
| Doss/2005 |  |  |  |  |  |
| Doss/2011 |  |  |  |  |  |
| Elhamamsy/2010 | 6 |  |  | 6 |  |
| Elhamamsy/2022 |  |  |  |  |  |
| Gofus/2022 |  |  |  |  |  |
| Grocott-Mason/2000 |  |  |  |  |  |
| Heuvelman/2013 |  |  |  |  |  |
| Jaggers/1998 |  |  |  |  |  |
| Klieverik/2008 |  |  |  |  |  |
| Knott-Craig/2000 |  |  |  |  |  |
| Laforest/2002 |  |  |  |  |  |
| Mazine/2016 |  |  |  |  |  |
| Mazine/2022 |  |  |  |  |  |
| Mokhles/2011 |  |  |  |  |  |
| Polito/2021/ |  |  |  |  |  |
| Santini/1997 |  |  |  |  |  |
| Sharabiani/2016 |  |  |  |  |  |
| Zacek/2016 |  |  |  |  |  |

|  | **Prior Cardiac Surgery (%)** | | | | |
| --- | --- | --- | --- | --- | --- |
|  | **Ross** | **MV** | **BV** | **Homograft** | **Ozaki** |
| **Author/Year** |  |  |  |  |  |
| Akhyari/2005 | 11.11 | 10 |  |  |  |
| Andreas/2014 | 9 | 15 |  |  |  |
| Bouhout/2017 |  |  |  |  |  |
| Buratto/2018 | 9 | 8 |  |  |  |
| Choudhary/1998 |  |  |  |  |  |
| Dagenais/2005 |  |  |  |  |  |
| Doss/2005 |  |  |  |  |  |
| Doss/2011 |  |  |  |  |  |
| Elhamamsy/2010 | 42 |  |  | 44 |  |
| Elhamamsy/2022 |  |  |  |  |  |
| Gofus/2022 | 8.6 | 9.6 |  |  |  |
| Grocott-Mason/2000 |  |  |  |  |  |
| Heuvelman/2013 |  |  |  |  |  |
| Jaggers/1998 |  |  |  |  |  |
| Klieverik/2008 | 27 |  |  | 20 |  |
| Knott-Craig/2000 |  |  |  |  |  |
| Laforest/2002 |  |  |  |  |  |
| Mazine/2016 | 41 | 45 |  |  |  |
| Mazine/2022 | 9 |  | 18 |  |  |
| Mokhles/2011 | 2.4 | 4 |  |  |  |
| Polito/2021/ | 43.8 |  |  |  | 22.8 |
| Santini/1997 |  |  |  |  |  |
| Sharabiani/2016 |  |  |  |  |  |
| Zacek/2016 |  |  |  |  |  |

|  | **Coronary Artery Disease (%)** | | | | |
| --- | --- | --- | --- | --- | --- |
|  | **Ross** | **MV** | **BV** | **Homograft** | **Ozaki** |
| **Author/Year** |  |  |  |  |  |
| Akhyari/2005 | 0 | 5 |  |  |  |
| Andreas/2014 |  |  |  |  |  |
| Bouhout/2017 | 1 | 3 |  |  |  |
| Buratto/2018 |  |  |  |  |  |
| Choudhary/1998 |  |  |  |  |  |
| Dagenais/2005 |  |  |  |  |  |
| Doss/2005 |  |  |  |  |  |
| Doss/2011 |  |  |  |  |  |
| Elhamamsy/2010 |  |  |  |  |  |
| Elhamamsy/2022 |  |  |  |  |  |
| Gofus/2022 | 6.5 | 7.6 |  |  |  |
| Grocott-Mason/2000 |  |  |  |  |  |
| Heuvelman/2013 |  |  |  |  |  |
| Jaggers/1998 | 0 | 0 |  |  |  |
| Klieverik/2008 |  |  |  |  |  |
| Knott-Craig/2000 |  |  |  |  |  |
| Laforest/2002 |  |  |  |  |  |
| Mazine/2016 |  |  |  |  |  |
| Mazine/2022 |  |  |  |  |  |
| Mokhles/2011 |  |  |  |  |  |
| Polito/2021/ |  |  |  |  |  |
| Santini/1997 |  |  |  |  |  |
| Sharabiani/2016 |  |  |  |  |  |
| Zacek/2016 |  |  |  |  |  |

|  | **Prior MI (%)** | | | | |
| --- | --- | --- | --- | --- | --- |
|  | **Ross** | **MV** | **BV** | **Homograft** | **Ozaki** |
| **Author/Year** |  |  |  |  |  |
| Akhyari/2005 |  |  |  |  |  |
| Andreas/2014 |  |  |  |  |  |
| Bouhout/2017 |  |  |  |  |  |
| Buratto/2018 | 0 | 0 |  |  |  |
| Choudhary/1998 |  |  |  |  |  |
| Dagenais/2005 |  |  |  |  |  |
| Doss/2005 |  |  |  |  |  |
| Doss/2011 |  |  |  |  |  |
| Elhamamsy/2010 |  |  |  |  |  |
| Elhamamsy/2022 |  |  |  |  |  |
| Gofus/2022 |  |  |  |  |  |
| Grocott-Mason/2000 |  | 17.8 | 12.8 | 6.3 |  |
| Heuvelman/2013 |  |  |  |  |  |
| Jaggers/1998 |  |  |  |  |  |
| Klieverik/2008 |  |  |  |  |  |
| Knott-Craig/2000 |  |  |  |  |  |
| Laforest/2002 |  |  |  |  |  |
| Mazine/2016 | 5 | 2 |  |  |  |
| Mazine/2022 |  |  |  |  |  |
| Mokhles/2011 |  |  |  |  |  |
| Polito/2021/ |  |  |  |  |  |
| Santini/1997 |  |  |  |  |  |
| Sharabiani/2016 |  |  |  |  |  |
| Zacek/2016 |  |  |  |  |  |

|  | **Left Ventricular Ejection Fraction, LVEF mean (SD), median / range** | | | | | | | | | |
| --- | --- | --- | --- | --- | --- | --- | --- | --- | --- | --- |
|  | **Ross** | | **MV** | | **BV** | | **Homograft** | | **Ozaki** | |
| **Author/Year** | **Mean** | **SD** | **Mean** | **SD** | **Mean** | **SD** | **Mean** | **SD** | **Mean** | **SD** |
| Akhyari/2005 | 65.4 | 6.7 | 52.2 | 16.2 |  |  |  |  |  |  |
| Andreas/2014 |  |  |  |  |  |  |  |  |  |  |
| Bouhout/2017 |  |  |  |  |  |  |  |  |  |  |
| Buratto/2018 |  |  |  |  |  |  |  |  |  |  |
| Choudhary/1998 |  |  |  |  |  |  |  |  |  |  |
| Dagenais/2005 |  |  |  |  |  |  |  |  |  |  |
| Doss/2005 | 66.3 | 7.9 | 67.2 | 6.8 |  |  |  |  |  |  |
| Doss/2011 |  |  |  |  |  |  |  |  |  |  |
| Elhamamsy/2010 |  |  |  |  |  |  |  |  |  |  |
| Elhamamsy/2022 |  |  |  |  |  |  |  |  |  |  |
| Gofus/2022 | 61.0 | (55.0–65.0) | 60.0 | (55.0–65.0) |  |  |  |  |  |  |
| Grocott-Mason/2000 |  |  |  |  |  |  |  |  |  |  |
| Heuvelman/2013 |  |  |  |  |  |  |  |  |  |  |
| Jaggers/1998 |  |  |  |  |  |  |  |  |  |  |
| Klieverik/2008 |  |  |  |  |  |  |  |  |  |  |
| Knott-Craig/2000 |  |  |  |  |  |  |  |  |  |  |
| Laforest/2002 |  |  |  |  |  |  |  |  |  |  |
| Mazine/2016 |  |  |  |  |  |  |  |  |  |  |
| Mazine/2022 |  |  |  |  |  |  |  |  |  |  |
| Mokhles/2011 | 64.0 | 11.2 | 65.6 | 14.2 |  |  |  |  |  |  |
| Polito/2021/ |  |  |  |  |  |  |  |  |  |  |
| Santini/1997 | 66 | 12 |  |  |  |  | 65 | 23 |  |  |
| Sharabiani/2016 |  |  |  |  |  |  |  |  |  |  |
| Zacek/2016 | 65 | 8 | 65 | 6 |  |  |  |  |  |  |

|  | **Pacemaker Implantation (%)** | | | | |
| --- | --- | --- | --- | --- | --- |
|  | **Ross** | **MV** | **BV** | **Homograft** | **Ozaki** |
| **Author/Year** |  |  |  |  |  |
| Akhyari/2005 |  |  |  |  |  |
| Andreas/2014 |  |  |  |  |  |
| Bouhout/2017 |  |  |  |  |  |
| Buratto/2018 |  |  |  |  |  |
| Choudhary/1998 |  |  |  |  |  |
| Dagenais/2005 |  |  |  |  |  |
| Doss/2005 |  |  |  |  |  |
| Doss/2011 |  |  |  |  |  |
| Elhamamsy/2010 | 4 |  |  | 1 |  |
| Elhamamsy/2022 |  |  |  |  |  |
| Gofus/2022 | 0.7 | 0.7 |  |  |  |
| Grocott-Mason/2000 |  |  |  |  |  |
| Heuvelman/2013 |  |  |  |  |  |
| Jaggers/1998 |  |  |  |  |  |
| Klieverik/2008 |  |  |  |  |  |
| Knott-Craig/2000 |  |  |  |  |  |
| Laforest/2002 |  |  |  |  |  |
| Mazine/2016 |  |  |  |  |  |
| Mazine/2022 | 2 |  | 0 |  |  |
| Mokhles/2011 |  |  |  |  |  |
| Polito/2021/ |  |  |  |  |  |
| Santini/1997 |  |  |  |  |  |
| Sharabiani/2016 |  |  |  |  |  |
| Zacek/2016 |  |  |  |  |  |

|  | **Liver disease / Cirrhosis (%)** | | | | |
| --- | --- | --- | --- | --- | --- |
|  | **Ross** | **MV** | **BV** | **Homograft** | **Ozaki** |
| **Author/Year** |  |  |  |  |  |
| Akhyari/2005 |  |  |  |  |  |
| Andreas/2014 |  |  |  |  |  |
| Bouhout/2017 |  |  |  |  |  |
| Buratto/2018 |  |  |  |  |  |
| Choudhary/1998 |  |  |  |  |  |
| Dagenais/2005 |  |  |  |  |  |
| Doss/2005 |  |  |  |  |  |
| Doss/2011 |  |  |  |  |  |
| Elhamamsy/2010 |  |  |  |  |  |
| Elhamamsy/2022 | 1 | 1 | 1 |  |  |
| Gofus/2022 |  |  |  |  |  |
| Grocott-Mason/2000 |  |  |  |  |  |
| Heuvelman/2013 |  |  |  |  |  |
| Jaggers/1998 |  |  |  |  |  |
| Klieverik/2008 |  |  |  |  |  |
| Knott-Craig/2000 |  |  |  |  |  |
| Laforest/2002 |  |  |  |  |  |
| Mazine/2016 |  |  |  |  |  |
| Mazine/2022 |  |  |  |  |  |
| Mokhles/2011 |  |  |  |  |  |
| Polito/2021/ |  |  |  |  |  |
| Santini/1997 |  |  |  |  |  |
| Sharabiani/2016 |  |  |  |  |  |
| Zacek/2016 |  |  |  |  |  |

|  | **Aortic stenosis (%)** | | | | |
| --- | --- | --- | --- | --- | --- |
|  | **Ross** | **MV** | **BV** | **Homograft** | **Ozaki** |
| **Author/Year** |  |  |  |  |  |
| Akhyari/2005 | 5.56 | 15 |  |  |  |
| Andreas/2014 | 33 | 42 |  |  |  |
| Bouhout/2017 | 69 | 70 |  |  |  |
| Buratto/2018 | 65 | 68 |  |  |  |
| Choudhary/1998 | 19.8 | 23.7 |  |  |  |
| Dagenais/2005 |  |  |  |  |  |
| Doss/2005 |  |  |  |  |  |
| Doss/2011 |  |  |  |  |  |
| Elhamamsy/2010 |  |  |  |  |  |
| Elhamamsy/2022 |  |  |  |  |  |
| Gofus/2022 |  |  |  |  |  |
| Grocott-Mason/2000 |  | 73.3 | 68.1 | 54.8 |  |
| Heuvelman/2013 | 56 | 0 |  | 31 |  |
| Jaggers/1998 | 68 | 70 |  |  |  |
| Klieverik/2008 | 38 |  |  | 29 |  |
| Knott-Craig/2000 |  |  |  |  |  |
| Laforest/2002 | 47 |  |  | 32 |  |
| Mazine/2016 | 119 | 70 |  |  |  |
| Mazine/2022 | 52 |  | 44 |  |  |
| Mokhles/2011 |  |  |  |  |  |
| Polito/2021/ |  |  |  |  |  |
| Santini/1997 | 58 |  |  | 59 |  |
| Sharabiani/2016 | 37.3 | 26.1 | 15.3 | 15.1 |  |
| Zacek/2016 | 36 | 45 |  |  |  |

|  | **Aortic regurgitation (%)** | | | | |
| --- | --- | --- | --- | --- | --- |
|  | **Ross** | **MV** | **BV** | **Homograft** | **Ozaki** |
| **Author/Year** |  |  |  |  |  |
| Akhyari/2005 | 22.22 | 50 |  |  |  |
| Andreas/2014 | 42 | 39 |  |  |  |
| Bouhout/2017 | 10 | 17 |  |  |  |
| Buratto/2018 | 55 | 52 |  |  |  |
| Choudhary/1998 | 55.2 | 46.2 |  |  |  |
| Dagenais/2005 |  |  |  |  |  |
| Doss/2005 |  |  |  |  |  |
| Doss/2011 |  |  |  |  |  |
| Elhamamsy/2010 |  |  |  |  |  |
| Elhamamsy/2022 |  |  |  |  |  |
| Gofus/2022 |  |  |  |  |  |
| Grocott-Mason/2000 |  | 12.2 | 10.6 | 26.5 |  |
| Heuvelman/2013 | 17 | 55 |  | 46 |  |
| Jaggers/1998 | 68 | 70 |  |  |  |
| Klieverik/2008 | 22 |  |  | 39 |  |
| Knott-Craig/2000 |  |  |  |  |  |
| Laforest/2002 | 15 |  |  | 47 |  |
| Mazine/2016 |  |  |  |  |  |
| Mazine/2022 |  |  |  |  |  |
| Mokhles/2011 |  |  |  |  |  |
| Polito/2021/ |  |  |  |  |  |
| Santini/1997 |  |  |  |  |  |
| Sharabiani/2016 | 23 | 31.9 | 35.6 | 32.1 |  |
| Zacek/2016 | 86 | 79 |  |  |  |

|  | **Diabetes (%)** | | | | |
| --- | --- | --- | --- | --- | --- |
|  | **Ross** | **MV** | **BV** | **Homograft** | **Ozaki** |
| **Author/Year** |  |  |  |  |  |
| Akhyari/2005 | 0 | 5 |  |  |  |
| Andreas/2014 |  |  |  |  |  |
| Bouhout/2017 | 7 | 9 |  |  |  |
| Buratto/2018 | 2 | 0 |  |  |  |
| Choudhary/1998 |  |  |  |  |  |
| Dagenais/2005 | 6.6 |  | 16.31 | 7.4 |  |
| Doss/2005 |  |  |  |  |  |
| Doss/2011 |  |  |  |  |  |
| Elhamamsy/2010 | 1 |  |  | 2 |  |
| Elhamamsy/2022 | 0.2 | 0 | 0.2 |  |  |
| Gofus/2022 | 3.7 | 6.5 |  |  |  |
| Grocott-Mason/2000 |  |  |  |  |  |
| Heuvelman/2013 |  |  |  |  |  |
| Jaggers/1998 |  |  |  |  |  |
| Klieverik/2008 |  |  |  |  |  |
| Knott-Craig/2000 |  |  |  |  |  |
| Laforest/2002 |  |  |  |  |  |
| Mazine/2016 | 3 | 5 |  |  |  |
| Mazine/2022 | 3 |  | 2 |  |  |
| Mokhles/2011 | 4.3 | 3.6 |  |  |  |
| Polito/2021/ |  |  |  |  |  |
| Santini/1997 |  |  |  |  |  |
| Sharabiani/2016 |  |  |  |  |  |
| Zacek/2016 | 0 | 0 |  |  |  |

**Supplementary Table 4.** Operative details of the included studies.

|  | **Procedure time (mins), mean (SD)** | | | | | | | | | |
| --- | --- | --- | --- | --- | --- | --- | --- | --- | --- | --- |
|  | **Ross** | | **MV** | | **BV** | | **Homograft** | | **Ozaki** | |
| **Author/Year** | **Mean** | **SD** | **Mean** | **SD** | **Mean** | **SD** | **Mean** | **SD** | **Mean** | **SD** |
| Akhyari/2005 |  |  |  |  |  |  |  |  |  |  |
| Andreas/2014 |  |  |  |  |  |  |  |  |  |  |
| Bouhout/2017 |  |  |  |  |  |  |  |  |  |  |
| Buratto/2018 |  |  |  |  |  |  |  |  |  |  |
| Choudhary/1998 |  |  |  |  |  |  |  |  |  |  |
| Dagenais/2005 |  |  |  |  |  |  |  |  |  |  |
| Doss/2005 |  |  |  |  |  |  |  |  |  |  |
| Doss/2011 |  |  |  |  |  |  |  |  |  |  |
| Elhamamsy/2010 |  |  |  |  |  |  |  |  |  |  |
| Elhamamsy/2022 |  |  |  |  |  |  |  |  |  |  |
| Gofus/2022 |  |  |  |  |  |  |  |  |  |  |
| Grocott-Mason/2000 |  |  |  |  |  |  |  |  |  |  |
| Heuvelman/2013 |  |  |  |  |  |  |  |  |  |  |
| Jaggers/1998 |  |  |  |  |  |  |  |  |  |  |
| Klieverik/2008 |  |  |  |  |  |  |  |  |  |  |
| Knott-Craig/2000 |  |  |  |  |  |  |  |  |  |  |
| Laforest/2002 |  |  |  |  |  |  |  |  |  |  |
| Mazine/2016 |  |  |  |  |  |  |  |  |  |  |
| Mazine/2022 |  |  |  |  |  |  |  |  |  |  |
| Mokhles/2011 |  |  |  |  |  |  |  |  |  |  |
| Polito/2021/ | 186 | (177-229) |  |  |  |  |  |  | 142 | (117-172) |
| Santini/1997 |  |  |  |  |  |  |  |  |  |  |
| Sharabiani/2016 |  |  |  |  |  |  |  |  |  |  |
| Zacek/2016 |  |  |  |  |  |  |  |  |  |  |

|  | **Cardiopulmonary bypass time (min), mean (SD)** | | | | | | | | | |
| --- | --- | --- | --- | --- | --- | --- | --- | --- | --- | --- |
|  | **Ross** | | **MV** | | **BV** | | **Homograft** | | **Ozaki** | |
| **Author/Year** | **Mean** | **SD** | **Mean** | **SD** | **Mean** | **SD** | **Mean** | **SD** | **Mean** | **SD** |
| Akhyari/2005 | 181.3 | 27.9 | 116.6 | 16.6 |  |  |  |  |  |  |
| Andreas/2014 |  |  |  |  |  |  |  |  |  |  |
| Bouhout/2017 | 212 | 42 | 91 | 42 |  |  |  |  |  |  |
| Buratto/2018 | 199 | 24 | 74 | 62 |  |  |  |  |  |  |
| Choudhary/1998 | 147.6 | (123 to 220) |  |  |  |  | 83.4 | 12.6 |  |  |
| Dagenais/2005 | 146 | 33.7 |  |  | 112.6 | 32.4 | 124.2 | 33.8 |  |  |
| Doss/2005 | 141 | 37 | 102 | 23 |  |  |  |  |  |  |
| Doss/2011 | 141 | 37 | 75 | 19 |  |  |  |  |  |  |
| Elhamamsy/2010 | 163 | 37 |  |  |  |  | 117 | 49 |  |  |
| Elhamamsy/2022 |  |  |  |  |  |  |  |  |  |  |
| Gofus/2022 | 193.0 | (171.0–217.5) | 95.0 | (72.0–120.0) |  |  |  |  |  |  |
| Grocott-Mason/2000 |  |  | 111 | 38 | 120 | 40 | 103 | 38 |  |  |
| Heuvelman/2013 |  |  |  |  |  |  |  |  |  |  |
| Jaggers/1998 |  |  |  |  |  |  |  |  |  |  |
| Klieverik/2008 | 206 | 76 |  |  |  |  | 165 | 42 |  |  |
| Knott-Craig/2000 |  |  |  |  |  |  |  |  |  |  |
| Laforest/2002 |  |  |  |  |  |  |  |  |  |  |
| Mazine/2016 | 144 | (133-156) | 92 | (73-123) |  |  |  |  |  |  |
| Mazine/2022 | 146 | (135-160) |  |  | 96 | (80-117) |  |  |  |  |
| Mokhles/2011 |  |  |  |  |  |  |  |  |  |  |
| Polito/2021/ |  |  |  |  |  |  |  |  |  |  |
| Santini/1997 | 151 | 31 |  |  |  |  | 113 | 29 |  |  |
| Sharabiani/2016 | 181.3 | 27.9 | 116.6 | 16.6 |  |  |  |  |  |  |
| Zacek/2016 |  |  |  |  |  |  |  |  |  |  |

|  | **Valve size, mean (SD) , median / IQR** | | | | | | | | | |
| --- | --- | --- | --- | --- | --- | --- | --- | --- | --- | --- |
|  | **Ross** | | **MV** | | **BV** | | **Homograft** | | **Ozaki** | |
| **Author/Year** | **Mean** | **SD** | **Mean** | **SD** | **Mean** | **SD** | **Mean** | **SD** | **Mean** | **SD** |
| Akhyari/2005 |  |  | 25.6 | 2.2 |  |  |  |  |  |  |
| Andreas/2014 | 27 | 2 | 24 | 2 |  |  |  |  |  |  |
| Bouhout/2017 |  |  |  |  |  |  |  |  |  |  |
| Buratto/2018 |  |  |  |  |  |  |  |  |  |  |
| Choudhary/1998 |  |  |  |  |  |  |  |  |  |  |
| Dagenais/2005 |  |  |  |  |  |  |  |  |  |  |
| Doss/2005 | 24.9 | 2.3 | 24.2 | 1.7 |  |  |  |  |  |  |
| Doss/2011 | 24.9 | 2.3 | 24.2 | 1.7 |  |  |  |  |  |  |
| Elhamamsy/2010 |  |  |  |  |  |  |  |  |  |  |
| Elhamamsy/2022 |  |  |  |  |  |  |  |  |  |  |
| Gofus/2022 |  |  |  |  |  |  |  |  |  |  |
| Grocott-Mason/2000 |  |  | 24 | 2 | 23 | 2 | 23 | 2 |  |  |
| Heuvelman/2013 |  |  | 21 | (21-23) |  |  | 22 | (21-22) |  |  |
| Jaggers/1998 |  |  | 25 | 3.3 |  |  |  |  |  |  |
| Klieverik/2008 |  |  |  |  |  |  |  |  |  |  |
| Knott-Craig/2000 |  |  |  |  |  |  |  |  |  |  |
| Laforest/2002 |  |  |  |  |  |  |  |  |  |  |
| Mazine/2016 |  |  |  |  |  |  |  |  |  |  |
| Mazine/2022 |  |  |  |  |  |  |  |  |  |  |
| Mokhles/2011 |  |  |  |  |  |  |  |  |  |  |
| Polito/2021/ |  |  |  |  |  |  |  |  |  |  |
| Santini/1997 |  |  |  |  |  |  |  |  |  |  |
| Sharabiani/2016 |  |  | 25.6 | 2.2 |  |  |  |  |  |  |
| Zacek/2016 | 27 | 2 | 24 | 2 |  |  |  |  |  |  |

|  | **Volume blood loss (mL), mean (SD), median / IQR** | | | | | | | | | |
| --- | --- | --- | --- | --- | --- | --- | --- | --- | --- | --- |
|  | **Ross** | | **MV** | | **BV** | | **Homograft** | | **Ozaki** | |
| **Author/Year** | **Mean** | **SD** | **Mean** | **SD** | **Mean** | **SD** | **Mean** | **SD** | **Mean** | **SD** |
| Akhyari/2005 |  |  |  |  |  |  |  |  |  |  |
| Andreas/2014 |  |  |  |  |  |  |  |  |  |  |
| Bouhout/2017 |  |  |  |  |  |  |  |  |  |  |
| Buratto/2018 |  |  |  |  |  |  |  |  |  |  |
| Choudhary/1998 |  |  |  |  |  |  |  |  |  |  |
| Dagenais/2005 |  |  |  |  |  |  |  |  |  |  |
| Doss/2005 |  |  |  |  |  |  |  |  |  |  |
| Doss/2011 |  |  |  |  |  |  |  |  |  |  |
| Elhamamsy/2010 |  |  |  |  |  |  |  |  |  |  |
| Elhamamsy/2022 |  |  |  |  |  |  |  |  |  |  |
| Gofus/2022 | 530 | (395-755) | 510 | (370-740) |  |  |  |  |  |  |
| Grocott-Mason/2000 |  |  |  |  |  |  |  |  |  |  |
| Heuvelman/2013 |  |  |  |  |  |  |  |  |  |  |
| Jaggers/1998 |  |  |  |  |  |  |  |  |  |  |
| Klieverik/2008 |  |  |  |  |  |  |  |  |  |  |
| Knott-Craig/2000 |  |  |  |  |  |  |  |  |  |  |
| Laforest/2002 |  |  |  |  |  |  |  |  |  |  |
| Mazine/2016 |  |  |  |  |  |  |  |  |  |  |
| Mazine/2022 |  |  |  |  |  |  |  |  |  |  |
| Mokhles/2011 |  |  |  |  |  |  |  |  |  |  |
| Polito/2021/ |  |  |  |  |  |  |  |  |  |  |
| Santini/1997 | 543 | 404 |  |  |  |  | 471 | 347 |  |  |
| Sharabiani/2016 |  |  |  |  |  |  |  |  |  |  |
| Zacek/2016 |  |  |  |  |  |  |  |  |  |  |

|  | **Emergent Operation (%)** | | | | |
| --- | --- | --- | --- | --- | --- |
|  | **Ross** | **MV** | **BV** | **Homograft** | **Ozaki** |
| **Author/Year** |  |  |  |  |  |
| Akhyari/2005 |  |  |  |  |  |
| Andreas/2014 |  |  |  |  |  |
| Bouhout/2017 |  |  |  |  |  |
| Buratto/2018 |  |  |  |  |  |
| Choudhary/1998 |  |  |  |  |  |
| Dagenais/2005 |  |  |  |  |  |
| Doss/2005 |  |  |  |  |  |
| Doss/2011 |  |  |  |  |  |
| Elhamamsy/2010 | 5 |  |  | 7 |  |
| Elhamamsy/2022 |  |  |  |  |  |
| Gofus/2022 |  |  |  |  |  |
| Grocott-Mason/2000 |  |  |  |  |  |
| Heuvelman/2013 |  |  |  |  |  |
| Jaggers/1998 |  |  |  |  |  |
| Klieverik/2008 |  |  |  |  |  |
| Knott-Craig/2000 |  |  |  |  |  |
| Laforest/2002 |  |  |  |  |  |
| Mazine/2016 |  |  |  |  |  |
| Mazine/2022 |  |  |  |  |  |
| Mokhles/2011 |  |  |  |  |  |
| Polito/2021/ |  |  |  |  |  |
| Santini/1997 |  |  |  |  |  |
| Sharabiani/2016 |  |  |  |  |  |
| Zacek/2016 |  |  |  |  |  |

|  | **Length of ICU stay (hours), mean (SD)** | | | | | | | | | |
| --- | --- | --- | --- | --- | --- | --- | --- | --- | --- | --- |
|  | **Ross** | | **MV** | | **BV** | | **Homograft** | | **Ozaki** | |
| **Author/Year** | **Mean** | **SD** | **Mean** | **SD** | **Mean** | **SD** | **Mean** | **SD** | **Mean** | **SD** |
| Akhyari/2005 | 28.8 | 16.8 | 28.8 | 9.6 |  |  |  |  |  |  |
| Andreas/2014 |  |  |  |  |  |  |  |  |  |  |
| Bouhout/2017 | 48 |  | 24 |  |  |  |  |  |  |  |
| Buratto/2018 |  |  |  |  |  |  |  |  |  |  |
| Choudhary/1998 |  |  |  |  |  |  |  |  |  |  |
| Dagenais/2005 |  |  |  |  |  |  |  |  |  |  |
| Doss/2005 |  |  |  |  |  |  |  |  |  |  |
| Doss/2011 |  |  |  |  |  |  |  |  |  |  |
| Elhamamsy/2010 | 24 | (24-336) |  |  |  |  | 24 | (0-206) |  |  |
| Elhamamsy/2022 |  |  |  |  |  |  |  |  |  |  |
| Gofus/2022 | 24.7 | (21.3–47.1) | 28.0 | (22.3–59.3) |  |  |  |  |  |  |
| Grocott-Mason/2000 |  |  |  |  |  |  |  |  |  |  |
| Heuvelman/2013 |  |  |  |  |  |  |  |  |  |  |
| Jaggers/1998 |  |  |  |  |  |  |  |  |  |  |
| Klieverik/2008 |  |  |  |  |  |  |  |  |  |  |
| Knott-Craig/2000 |  |  |  |  |  |  |  |  |  |  |
| Laforest/2002 |  |  |  |  |  |  |  |  |  |  |
| Mazine/2016 |  |  |  |  |  |  |  |  |  |  |
| Mazine/2022 |  |  |  |  |  |  |  |  |  |  |
| Mokhles/2011 |  |  |  |  |  |  |  |  |  |  |
| Polito/2021/ |  |  |  |  |  |  |  |  |  |  |
| Santini/1997 | 2 | 3.7 |  |  |  |  | 1.2 | 0.6 |  |  |
| Sharabiani/2016 |  |  | 28.8 | 9.6 |  |  |  |  |  |  |
| Zacek/2016 |  |  |  |  |  |  |  |  |  |  |

|  | **Length of index hospital stay (days), mean (SD)** | | | | | | | | | |
| --- | --- | --- | --- | --- | --- | --- | --- | --- | --- | --- |
|  | **Ross** | | **MV** | | **BV** | | **Homograft** | | **Ozaki** | |
| **Author/Year** | **Mean** | **SD** | **Mean** | **SD** | **Mean** | **SD** | **Mean** | **SD** | **Mean** | **SD** |
| Akhyari/2005 | 11.2 | 4.3 | 13.5 | 6.1 |  |  |  |  |  |  |
| Andreas/2014 |  |  |  |  |  |  |  |  |  |  |
| Bouhout/2017 | 3 |  | 2 |  |  |  |  |  |  |  |
| Buratto/2018 |  |  |  |  |  |  |  |  |  |  |
| Choudhary/1998 |  |  |  |  |  |  |  |  |  |  |
| Dagenais/2005 | 8 | 4.4 |  |  | 8.28 | 4.68 | 8.9 | 8.2 |  |  |
| Doss/2005 |  |  |  |  |  |  |  |  |  |  |
| Doss/2011 |  |  |  |  |  |  |  |  |  |  |
| Elhamamsy/2010 | 9 | (4-44) |  |  |  |  | 9 | (0-46) |  |  |
| Elhamamsy/2022 | 5 |  | 6 |  |  |  |  |  |  |  |
| Gofus/2022 | 10.0 | (8.0–13.0) | 9.0 | (7.0–13.0) |  |  |  |  |  |  |
| Grocott-Mason/2000 |  |  |  |  |  |  |  |  |  |  |
| Heuvelman/2013 |  |  |  |  |  |  |  |  |  |  |
| Jaggers/1998 | 5.9 | 2.1 | 8 | 1.85 |  |  |  |  |  |  |
| Klieverik/2008 | 11 | 4 |  |  |  |  | 13 | 10 |  |  |
| Knott-Craig/2000 |  |  |  |  |  |  |  |  |  |  |
| Laforest/2002 |  |  |  |  |  |  |  |  |  |  |
| Mazine/2016 |  |  |  |  |  |  |  |  |  |  |
| Mazine/2022 |  |  |  |  |  |  |  |  |  |  |
| Mokhles/2011 |  |  |  |  |  |  |  |  |  |  |
| Polito/2021/ | 8 | (7-14.5) |  |  |  |  |  |  | 8 | (7-11) |
| Santini/1997 | 12 | 6 |  |  |  |  | 9.5 | 3.2 |  |  |
| Sharabiani/2016 |  |  |  |  |  |  |  |  |  |  |
| Zacek/2016 |  |  |  |  |  |  |  |  |  |  |

**Supplementary Table 5.** Summary of critical appraisal of included observational studies using the Newcastle Ottawa Quality Assessment Scale for Cohort Studies.

| Study/Year | Selection | Comparability | Outcome |
| --- | --- | --- | --- |
| Akhyari/2005 | **** | - | *** |
| Andreas/2014 | **** | * | *** |
| Bouhout/2017 | **** | - | *** |
| Buratto/2018 | **** | * | *** |
| Choudhary/1998 | ** | - | ** |
| Dagenais/2005 | *** | - | *** |
| Doss/2011 | **** | - | *** |
| Elhamamsy/2010 | **** | - | *** |
| Elhamamsy/2022 | **** | - | *** |
| Gofus/2022 | **** | * | *** |
| Grocott-Mason/2000 | **** | - | *** |
| Heuvelman/2013 | **** | * | *** |
| Jaggers/1998 | *** | - | *** |
| Klieverik/2008 | **** | * | ** |
| Knott-Craig/2000 | **** | - | *** |
| Laforest/2002 | **** | * | ** |
| Mazine/2016 | **** | - | ** |
| Mazine/2022 | **** | - | *** |
| Mokhles/2011 | **** | * | *** |
| Polito/2021/ | **** | * | *** |
| Sharabiani/2016 | **** | - | *** |
| Zacek/2016 | *** | - | *** |

**Selection**

 1)        Representativeness of intervention cohort a) truly representative of the average in the community *; b) somewhat representative of the average; c) only selected group of users; d) no description of the derivation of the cohort.

2)        Selection of nonintervention cohort – a) drawn from same community as intervention cohort*; b) drawn from a different source; c) no description of the derivation of the nonexposed cohort.

3)        Ascertainment of exposure a) secure record*; b) structured interview*; c) written self-report; d) no description.

4)        Demonstration that outcome of interest was not present at start of study a) yes*; b) no.

**Comparability**

 1)       Comparability of cohorts on the basis of the design or analysis a) study controls for age, and gender*; b) study controls for any additional factor***.**

**Outcome**

 1)       Assessment of outcome a) independent blind assessment***;**b) record linkage*; c) self-report; d) no description.

2)       Was follow-up long enough for outcomes to occur a) yes*; b) no

3)       Adequacy of follow up of cohorts a) complete follow up***;**b) subjects lost to follow up unlikely to introduce bias < 20 % lost follow up***;**c) follow up rate < 80% and no description of those lost; d) no statement

**Supplementary Table 6.** The Cochrane Risk-of-Bias Tool for Randomized Trials (RoB 2).

| **Study details**   \| **Reference** \| Doss M, Wood JP, Martens S, Wimmer-Greinecker G, Moritz A. Do pulmonary autografts provide better outcomes than mechanical valves? A prospective randomized trial. Ann Thorac Surg. 2005;80(6):2194-2198. doi:10.1016/j.athoracsur.2005.06.006 \| \| --- \| --- \|   **Study design**   \| X \| Individually-randomized parallel-group trial \| \| --- \| --- \| \| □ \| Cluster-randomized parallel-group trial \| \| □ \| Individually randomized cross-over (or other matched) trial \|   **For the purposes of this assessment, the interventions being compared are defined as**   \| Experimental: \| Mechanical aortic valves \| Comparator: \| Pulmonary autograft \| \| --- \| --- \| --- \| --- \|  \| **Specify which outcome is being assessed for risk of bias** \| 30-day mortality, Long-term mortality \| \| --- \| --- \|  \| **Specify the numerical result being assessed.** In case of multiple alternative analyses being presented, specify the numeric result (e.g. RR = 1.52 (95% CI 0.83 to 2.77) and/or a reference (e.g. to a table, figure or paragraph) that uniquely defines the result being assessed. \| Manuscript results \| \| --- \| --- \|   **Is the review team’s aim for this result…?**   \| X \| to assess the effect of *assignment to intervention* (the ‘intention-to-treat’ effect) \| \| --- \| --- \| \| □ \| to assess the effect of *adhering to intervention* (the ‘per-protocol’ effect) \|   **If the aim is to assess the effect of *adhering to intervention***, select the deviations from intended intervention that should be addressed (at least one must be checked):  □ occurrence of non-protocol interventions  □ failures in implementing the intervention that could have affected the outcome  □ non-adherence to their assigned intervention by trial participants  **Which of the following sources were obtained to help inform the risk-of-bias assessment? (tick as many as apply)**  X Journal article(s) with results of the trial  □ Trial protocol  □ Statistical analysis plan (SAP)  □ Non-commercial trial registry record (e.g. ClinicalTrials.gov record)  □ Company-owned trial registry record (e.g. GSK Clinical Study Register record)  □ “Grey literature” (e.g. unpublished thesis)  □ Conference abstract(s) about the trial  □ Regulatory document (e.g. Clinical Study Report, Drug Approval Package)  □ Research ethics application  □ Grant database summary (e.g. NIH RePORTER or Research Councils UK Gateway to Research)  □ Personal communication with trialist  □ Personal communication with the sponsor |
| --- | --- | --- | --- | --- | --- | --- | --- | --- | --- | --- | --- | --- | --- | --- | --- | --- | --- | --- | --- | --- |

Risk of bias assessment

Responses underlined in green are potential markers for low risk of bias, and responses in red are potential markers for a risk of bias. Where questions relate only to sign posts to other questions, no formatting is used.

**Domain 1: Risk of bias arising from the randomization process**

| **Signalling questions** | **Comments** | **Response options** |
| --- | --- | --- |
| **1.1 Was the allocation sequence random?** |  | Y |
| **1.2 Was the allocation sequence concealed until participants were enrolled and assigned to interventions?** |  | Y |
| **1.3 Did baseline differences between intervention groups suggest a problem with the randomization process?** |  | PN |
| **Risk-of-bias judgement** |  | Low |
| Optional: What is the predicted direction of bias arising from the randomization process? |  | Unpredictable |

Domain 2: Risk of bias due to deviations from the intended interventions (*effect of assignment to intervention*)

| **Signalling questions** | **Comments** | **Response options** |
| --- | --- | --- |
| **2.1. Were participants aware of their assigned intervention during the trial?** |  | Y |
| **2.2. Were carers and people delivering the interventions aware of participants' assigned intervention during the trial?** |  | Y |
| **2.3. If Y/PY/NI to 2.1 or 2.2: Were there deviations from the intended intervention that arose because of the trial context?** |  | PY |
| **2.4 If Y/PY to 2.3: Were these deviations likely to have affected the outcome?** |  | PY |
| **2.5. If Y/PY/NI to 2.4: Were these deviations from intended intervention balanced between groups?** |  | PY |
| **2.6 Was an appropriate analysis used to estimate the effect of assignment to intervention?** |  | Y |
| **2.7 If N/PN/NI to 2.6: Was there potential for a substantial impact (on the result) of the failure to analyse participants in the group to which they were randomized?** |  |  |
| **Risk-of-bias judgement** |  | Some concerns |
| Optional: What is the predicted direction of bias due to deviations from intended interventions? |  | Unpredictable |

Domain 2: Risk of bias due to deviations from the intended interventions (*effect of adhering to intervention*)

| **Signalling questions** | **Comments** | **Response options** |
| --- | --- | --- |
| **2.1. Were participants aware of their assigned intervention during the trial?** |  | NI |
| **2.2. Were carers and people delivering the interventions aware of participants' assigned intervention during the trial?** |  | NI |
| **2.3. [If applicable:] If Y/PY/NI to 2.1 or 2.2: Were important non-protocol interventions balanced across intervention groups?** |  | PY |
| **2.4. [If applicable:] Were there failures in implementing the intervention that could have affected the outcome?** |  | NI |
| **2.5. [If applicable:] Was there non-adherence to the assigned intervention regimen that could have affected participants’ outcomes?** |  | Y |
| **2.6. If N/PN/NI to 2.3, or Y/PY/NI to 2.4 or 2.5: Was an appropriate analysis used to estimate the effect of adhering to the intervention?** |  | PY |
| **Risk-of-bias judgement** |  | Some concerns |
| Optional: What is the predicted direction of bias due to deviations from intended interventions? |  | Unpredictable |

Domain 3: Missing outcome data

| **Signalling questions** | **Comments** | **Response options** |
| --- | --- | --- |
| **3.1 Were data for this outcome available for all, or nearly all, participants randomized?** |  | Y |
| **3.2 If N/PN/NI to 3.1: Is there evidence that the result was not biased by missing outcome data?** |  | NA |
| **3.3 If N/PN to 3.2: Could missingness in the outcome depend on its true value?** |  | NA |
| **3.4 If Y/PY/NI to 3.3: Is it likely that missingness in the outcome depended on its true value?** |  | NA |
| **Risk-of-bias judgement** |  | Low |
| Optional: What is the predicted direction of bias due to missing outcome data? |  | Unpredictable |

Domain 4: Risk of bias in measurement of the outcome

| **Signalling questions** | **Comments** | **Response options** |
| --- | --- | --- |
| **4.1 Was the method of measuring the outcome inappropriate?** |  | N |
| **4.2 Could measurement or ascertainment of the outcome have differed between intervention groups?** |  | N |
| **4.3 If N/PN/NI to 4.1 and 4.2: Were outcome assessors aware of the intervention received by study participants?** |  | Y |
| **4.4 If Y/PY/NI to 4.3: Could assessment of the outcome have been influenced by knowledge of intervention received?** |  | N |
| **4.5 If Y/PY/NI to 4.4: Is it likely that assessment of the outcome was influenced by knowledge of intervention received?** |  | N |
| **Risk-of-bias judgement** |  | Low |
| Optional: What is the predicted direction of bias in measurement of the outcome? |  | Unpredictable |

Domain 5: Risk of bias in selection of the reported result

| **Signalling questions** | **Comments** | **Response options** |
| --- | --- | --- |
| **5.1 Were the data that produced this result analysed in accordance with a pre-specified analysis plan that was finalized before unblinded outcome data were available for analysis?** |  | PY |
| **Is the numerical result being assessed likely to have been selected, on the basis of the results, from...** |  |  |
| **5.2. ... multiple eligible outcome measurements (e.g. scales, definitions, time points) within the outcome domain?** |  | N |
| **5.3 ... multiple eligible analyses of the data?** |  | N |
| **Risk-of-bias judgement** |  | Low |
| Optional: What is the predicted direction of bias due to selection of the reported result? |  | Unpredictable |

Overall risk of bias

| **Risk-of-bias judgement** |  | Low |
| --- | --- | --- |
| Optional: What is the overall predicted direction of bias for this outcome? |  | Unpredictable |

| **Study details**   \| **Reference** \| Santini F, Luciani GB, Pessotto R, et al. Sostituzione valvolare aortica mediante autograft polmonare: esperienza dell'Università di Verona [Replacement of the aortic valve with a pulmonary autograft: experience at the University of Verona]. G Ital Cardiol. 1997;27(2):141-145. \| \| --- \| --- \|   **Study design**   \| X \| Individually-randomized parallel-group trial \| \| --- \| --- \| \| □ \| Cluster-randomized parallel-group trial \| \| □ \| Individually randomized cross-over (or other matched) trial \|   **For the purposes of this assessment, the interventions being compared are defined as**   \| Experimental: \| Homograft \| Comparator: \| Pulmonary autograft \| \| --- \| --- \| --- \| --- \|  \| **Specify which outcome is being assessed for risk of bias** \| 30-day mortality, Long-term mortality \| \| --- \| --- \|  \| **Specify the numerical result being assessed.** In case of multiple alternative analyses being presented, specify the numeric result (e.g. RR = 1.52 (95% CI 0.83 to 2.77) and/or a reference (e.g. to a table, figure or paragraph) that uniquely defines the result being assessed. \| Manuscript results \| \| --- \| --- \|   **Is the review team’s aim for this result…?**   \| X \| to assess the effect of *assignment to intervention* (the ‘intention-to-treat’ effect) \| \| --- \| --- \| \| □ \| to assess the effect of *adhering to intervention* (the ‘per-protocol’ effect) \|   **If the aim is to assess the effect of *adhering to intervention***, select the deviations from intended intervention that should be addressed (at least one must be checked):  □ occurrence of non-protocol interventions  □ failures in implementing the intervention that could have affected the outcome  □ non-adherence to their assigned intervention by trial participants  **Which of the following sources were obtained to help inform the risk-of-bias assessment? (tick as many as apply)**  X Journal article(s) with results of the trial  □ Trial protocol  □ Statistical analysis plan (SAP)  □ Non-commercial trial registry record (e.g. ClinicalTrials.gov record)  □ Company-owned trial registry record (e.g. GSK Clinical Study Register record)  □ “Grey literature” (e.g. unpublished thesis)  □ Conference abstract(s) about the trial  □ Regulatory document (e.g. Clinical Study Report, Drug Approval Package)  □ Research ethics application  □ Grant database summary (e.g. NIH RePORTER or Research Councils UK Gateway to Research)  □ Personal communication with trialist  □ Personal communication with the sponsor |
| --- | --- | --- | --- | --- | --- | --- | --- | --- | --- | --- | --- | --- | --- | --- | --- | --- | --- | --- | --- | --- |

Risk of bias assessment

Responses underlined in green are potential markers for low risk of bias, and responses in red are potential markers for a risk of bias. Where questions relate only to sign posts to other questions, no formatting is used.

**Domain 1: Risk of bias arising from the randomization process**

| **Signalling questions** | **Comments** | **Response options** |
| --- | --- | --- |
| **1.1 Was the allocation sequence random?** |  | Y |
| **1.2 Was the allocation sequence concealed until participants were enrolled and assigned to interventions?** |  | Y |
| **1.3 Did baseline differences between intervention groups suggest a problem with the randomization process?** |  | PN |
| **Risk-of-bias judgement** |  | Low |
| Optional: What is the predicted direction of bias arising from the randomization process? |  | Unpredictable |

Domain 2: Risk of bias due to deviations from the intended interventions (*effect of assignment to intervention*)

| **Signalling questions** | **Comments** | **Response options** |
| --- | --- | --- |
| **2.1. Were participants aware of their assigned intervention during the trial?** |  | Y |
| **2.2. Were carers and people delivering the interventions aware of participants' assigned intervention during the trial?** |  | Y |
| **2.3. If Y/PY/NI to 2.1 or 2.2: Were there deviations from the intended intervention that arose because of the trial context?** |  | PY |
| **2.4 If Y/PY to 2.3: Were these deviations likely to have affected the outcome?** |  | PY |
| **2.5. If Y/PY/NI to 2.4: Were these deviations from intended intervention balanced between groups?** |  | PY |
| **2.6 Was an appropriate analysis used to estimate the effect of assignment to intervention?** |  | Y |
| **2.7 If N/PN/NI to 2.6: Was there potential for a substantial impact (on the result) of the failure to analyse participants in the group to which they were randomized?** |  |  |
| **Risk-of-bias judgement** |  | Some concerns |
| Optional: What is the predicted direction of bias due to deviations from intended interventions? |  | Unpredictable |

Domain 2: Risk of bias due to deviations from the intended interventions (*effect of adhering to intervention*)

| **Signalling questions** | **Comments** | **Response options** |
| --- | --- | --- |
| **2.1. Were participants aware of their assigned intervention during the trial?** |  | NI |
| **2.2. Were carers and people delivering the interventions aware of participants' assigned intervention during the trial?** |  | NI |
| **2.3. [If applicable:] If Y/PY/NI to 2.1 or 2.2: Were important non-protocol interventions balanced across intervention groups?** |  | PY |
| **2.4. [If applicable:] Were there failures in implementing the intervention that could have affected the outcome?** |  | NI |
| **2.5. [If applicable:] Was there non-adherence to the assigned intervention regimen that could have affected participants’ outcomes?** |  | Y |
| **2.6. If N/PN/NI to 2.3, or Y/PY/NI to 2.4 or 2.5: Was an appropriate analysis used to estimate the effect of adhering to the intervention?** |  | PY |
| **Risk-of-bias judgement** |  | Some concerns |
| Optional: What is the predicted direction of bias due to deviations from intended interventions? |  | Unpredictable |

Domain 3: Missing outcome data

| **Signalling questions** | **Comments** | **Response options** |
| --- | --- | --- |
| **3.1 Were data for this outcome available for all, or nearly all, participants randomized?** |  | Y |
| **3.2 If N/PN/NI to 3.1: Is there evidence that the result was not biased by missing outcome data?** |  | NA |
| **3.3 If N/PN to 3.2: Could missingness in the outcome depend on its true value?** |  | NA |
| **3.4 If Y/PY/NI to 3.3: Is it likely that missingness in the outcome depended on its true value?** |  | NA |
| **Risk-of-bias judgement** |  | Low |
| Optional: What is the predicted direction of bias due to missing outcome data? |  | Unpredictable |

Domain 4: Risk of bias in measurement of the outcome

| **Signalling questions** | **Comments** | **Response options** |
| --- | --- | --- |
| **4.1 Was the method of measuring the outcome inappropriate?** |  | N |
| **4.2 Could measurement or ascertainment of the outcome have differed between intervention groups?** |  | N |
| **4.3 If N/PN/NI to 4.1 and 4.2: Were outcome assessors aware of the intervention received by study participants?** |  | Y |
| **4.4 If Y/PY/NI to 4.3: Could assessment of the outcome have been influenced by knowledge of intervention received?** |  | N |
| **4.5 If Y/PY/NI to 4.4: Is it likely that assessment of the outcome was influenced by knowledge of intervention received?** |  | N |
| **Risk-of-bias judgement** |  | Low |
| Optional: What is the predicted direction of bias in measurement of the outcome? |  | Unpredictable |

Domain 5: Risk of bias in selection of the reported result

| **Signalling questions** | **Comments** | **Response options** |
| --- | --- | --- |
| **5.1 Were the data that produced this result analysed in accordance with a pre-specified analysis plan that was finalized before unblinded outcome data were available for analysis?** |  | PY |
| **Is the numerical result being assessed likely to have been selected, on the basis of the results, from...** |  |  |
| **5.2. ... multiple eligible outcome measurements (e.g. scales, definitions, time points) within the outcome domain?** |  | N |
| **5.3 ... multiple eligible analyses of the data?** |  | N |
| **Risk-of-bias judgement** |  | Low |
| Optional: What is the predicted direction of bias due to selection of the reported result? |  | Unpredictable |

Overall risk of bias

| **Risk-of-bias judgement** |  | Low |
| --- | --- | --- |
| Optional: What is the overall predicted direction of bias for this outcome? |  | Unpredictable |

Supplementary table 7: Rank table of outcome 30 days mortality

|  | V2 | V3 | V4 | V5 |
| --- | --- | --- | --- | --- |
| 1 | Homograft | | | |
| 2 | 0.99 [0.54; 1.82] | Ross | | |
| 3 | 0.53 [0.30; 0.93] | 0.53 [0.29; 0.96] | MV | |
| 4 | 0.42 [0.23; 0.76] | 0.42 [0.21; 0.83] | 0.79 [0.44; 1.44] | BV |

Supplementary table 8: Rank table of outcome 30 days stroke

|  | V1 | V2 | V3 | V4 | V5 |
| --- | --- | --- | --- | --- | --- |
| 1 | Ross |  |  |  |  |
| 2 | 1.00 [0.06; 15.78] | BV |  |  |  |
| 3 | 0.56 [0.02; 13.52] | 0.56 [0.01; 37.81] | Homograft | |  |
| 4 | 0.45 [0.02; 10.47] | 0.45 [0.01; 29.64] | 0.81 [0.01; 71.08] | Ozaki |  |
| 5 | 0.12 [0.02; 0.64] | 0.12 [0.00; 3.01] | 0.21 [0.01; 7.79] | 0.26 [0.01; 9.18] | MV |

Supplementary table 9: Rank table of outcome 30 days MI

|  | V1 | V2 | V3 | V4 |
| --- | --- | --- | --- | --- |
| 1 | BV | | | |
| 2 | 0.85 [0.11; 6.66] | MV | | |
| 3 | 0.84 [0.07; 10.88] | 0.99 [0.10; 9.57] | Homograft | |
| 4 | 0.50 [0.09; 2.67] | 0.59 [0.18; 1.93] | 0.59 [0.09; 4.12] | Ross |

Supplementary table 10: Rank table of outcome 30 days major bleeding

|  | V1 | V2 | V3 | V4 |
| --- | --- | --- | --- | --- |
| 1 | Homograft | |  |  |
| 2 | 0.35 [0.17; 0.71] | Ross | | |
| 3 | 0.15 [0.05; 0.49] | 0.43 [0.15; 1.24] | BV | |
| 4 | 0.08 [0.02; 0.23] | 0.22 [0.09; 0.52] | 0.50 [0.15; 1.65] | MV |

Supplementary table 11: Rank table of outcome long term mortality

|  | V1 | V2 | V3 | V4 | V5 |
| --- | --- | --- | --- | --- | --- |
| 1 | Ross | | | | |
| 2 | 0.72 [0.53; 0.98] | Homograft | | | |
| 3 | 0.69 [0.07; 7.13] | 0.95 [0.09; 10.06] | Ozaki | | |
| 4 | 0.53 [0.39; 0.72] | 0.73 [0.51; 1.04] | 0.77 [0.07; 8.15] | BV | |
| 5 | 0.52 [0.40; 0.66] | 0.71 [0.52; 0.97] | 0.75 [0.07; 7.87] | 0.97 [0.72; 1.31] | MV |

Supplementary table 12: Rank table of outcome long term stroke

|  | V1 | V2 | V3 | V4 |
| --- | --- | --- | --- | --- |
| 1 | Ross |  |  |  |
| 2 | 0.68 [0.31; 1.48] | MV |  |  |
| 3 | 0.67 [0.30; 1.50] | 0.98 [0.48; 2.01] | BV |  |
| 4 | 0.27 [0.01; 5.31] | 0.40 [0.02; 8.60] | 0.41 [0.02; 8.87] | Ozaki |

Supplementary table 13: Rank table of outcome long term re-intervention

|  | V1 | V2 | V3 | V4 | V5 |
| --- | --- | --- | --- | --- | --- |
| 1 | MV |  |  |  |  |
| 2 | 0.57 [0.26; 1.22] | Ross |  |  |  |
| 3 | 0.26 [0.02; 3.92] | 0.46 [0.03; 6.20] | Ozaki |  |  |
| 4 | 0.23 [0.08; 0.66] | 0.41 [0.18; 0.96] | 0.90 [0.06; 13.92] | Homograft | |
| 5 | 0.17 [0.06; 0.54] | 0.31 [0.11; 0.82] | 0.67 [0.04; 10.82] | 0.74 [0.23; 2.36] | BV |

Supplementary table 14: Summary of our outcomes

| **Author** | **Operative/30-day/in-hospital mortality (Number)** | | | | | **Operative/30-day/in-hospital stroke (Number)** | | | | | **Operative/30-day/in-hospital myocardial infarction(Number)** | | | | | **Post-operative major bleeding (number)** | | | | | **Reintervention (number)** | | | | | **Long term mortality (number)** | | | | | **Long term stroke (number)** | | | | |
| --- | --- | --- | --- | --- | --- | --- | --- | --- | --- | --- | --- | --- | --- | --- | --- | --- | --- | --- | --- | --- | --- | --- | --- | --- | --- | --- | --- | --- | --- | --- | --- | --- | --- | --- | --- |
|  | **Ross** | **MV** | **BV** | **Homograft** | **Ozaki** | **Ross** | **MV** | **BV** | **Homograft** | **Ozaki** | **Ross** | **MV** | **BV** | **Homograft** | **Ozaki** | **Ross** | **MV** | **BV** | **Homograft** | **Ozaki** | **Ross** | **MV** | **BV** | **Homograft** | **Ozaki** | **Ross** | **MV** | **BV** | **Homograft** | **Ozaki** | **Ross** | **MV** | **BV** | **Homograft** | **Ozaki** |
| Akhyari/2005 | 0 | 0 | NA | NA | NA | 0 | 0 | NA | NA | NA | 0 | 0 | NA | NA | NA | 0 | 0 | NA | NA | NA | 0 | 0 | NA | NA | NA | 0 | 1 | NA | NA | NA | 0 | 0 | NA | NA | NA |
| Andreas/2014 | 3 | 2 | NA | NA | NA | 1 | 10 | NA | NA | NA | NA | 1 | NA | NA | NA | 1 | 18 | NA | NA | NA | 20 | 10 | NA | NA | NA | 8 | 28 | NA | NA | NA | NA | NA | NA | NA | NA |
| Bouhout/2017 | 0 | 0 | NA | NA | NA | NA | NA | NA | NA | NA | 0 | 0 | NA | NA | NA | 3 | 6 | NA | NA | NA | NA | NA | NA | NA | NA | NA | NA | NA | NA | NA | NA | NA | NA | NA | NA |
| Buratto/2018 | 0 | 1 | NA | NA | NA | NA | NA | NA | NA | NA | NA | NA | NA | NA | NA | NA | NA | NA | NA | NA | NA | NA | NA | NA | NA | NA | NA | NA | NA | NA | NA | NA | NA | NA | NA |
| Choudhary/1998 | 7 | NA | NA | 7 | NA | NA | NA | NA | NA | NA | NA | NA | NA | NA | NA | 5 | NA | NA | 3 | NA | 9 | NA | NA | 4 | NA | 14 | NA | NA | 10 | NA | NA | NA | NA | NA | NA |
| Dagenais/2005 | 1 | NA | 5 | 2 | NA | NA | NA | NA | NA | NA | NA | NA | NA | NA | NA | NA | NA | NA | NA | NA | NA | NA | NA | NA | NA | NA | NA | NA | NA | NA | NA | NA | NA | NA | NA |
| Doss/2005 | 0 | 0 | NA | NA | NA | NA | NA | NA | NA | NA | NA | NA | NA | NA | NA | 0 | 3 | NA | NA | NA | 2 | 0 | NA | NA | NA | 2 | 1 | NA | NA | NA | 1 | 0 | NA | NA | NA |
| Doss/2011 | NA | NA | NA | NA | NA | NA | NA | NA | NA | NA | NA | NA | NA | NA | NA | NA | NA | NA | NA | NA | NA | NA | NA | NA | NA | 2 | 1 | NA | NA | NA | NA | NA | NA | NA | NA |
| Elhamamsy/2010 | 1 | NA | NA | 3 | NA | NA | NA | NA | NA | NA | NA | NA | NA | NA | NA | NA | NA | NA | NA | NA | NA | NA | NA | NA | NA | 3 | NA | NA | 5 | NA | NA | NA | NA | NA | NA |
| Elhamamsy/2022 | 1 | 3 | 3 | NA | NA | NA | NA | NA | NA | NA | NA | NA | NA | NA | NA | NA | NA | NA | NA | NA | NA | NA | NA | NA | NA | 30 | 50 | 53 | NA | NA | 9 | 14 | 14 | NA | NA |
| Gofus/2022 | 0 | 0 | NA | NA | NA | NA | NA | NA | NA | NA | 1 | 1 | NA | NA | NA | NA | NA | NA | NA | NA | NA | NA | NA | NA | NA | 19 | 19 | NA | NA | NA | NA | NA | NA | NA | NA |
| Grocott-Mason/2000 | NA | 10 | 8 | 19 | NA | NA | NA | NA | NA | NA | NA | NA | NA | NA | NA | NA | NA | NA | NA | NA | NA | NA | NA | NA | NA | NA | 43 | 19 | 133 | NA | NA | NA | NA | NA | NA |
| Heuvelman/2013 | 0 | 0 | NA | 0 | NA | NA | NA | NA | NA | NA | NA | NA | NA | NA | NA | NA | NA | NA | NA | NA | NA | NA | NA | NA | NA | NA | NA | NA | NA | NA | NA | NA | NA | NA | NA |
| Jaggers/1998 | 0 | 0 | NA | NA | NA | NA | NA | NA | NA | NA | 1 | 0 | NA | NA | NA | 0 | 1 | NA | NA | NA | 1 | 2 | NA | NA | NA | 0 | 2 | NA | NA | NA | NA | NA | NA | NA | NA |
| Klieverik/2008 | 2 | NA | NA | 0 | NA | 0 | NA | NA | 1 | NA | 2 | NA | NA | 2 | NA | 18 | NA | NA | 7 | NA | NA | NA | NA | NA | NA | 3 | NA | NA | 8 | NA | NA | NA | NA | NA | NA |
| Knott-Craig/2000 | NA | NA | NA | NA | NA | NA | NA | NA | NA | NA | NA | NA | NA | NA | NA | NA | NA | NA | NA | NA | 17 | NA | NA | 26 | NA | 33 | NA | NA | 31 | NA | NA | NA | NA | NA | NA |
| Laforest/2002 | NA | NA | NA | NA | NA | NA | NA | NA | NA | NA | NA | NA | NA | NA | NA | NA | NA | NA | NA | NA | 1 | NA | NA | 3 | NA | NA | NA | NA | NA | NA | NA | NA | NA | NA | NA |
| Mazine/2016 | 1 | 1 | NA | NA | NA | 0 | 3 | NA | NA | NA | 5 | 2 | NA | NA | NA | 0 | 27 | NA | NA | NA | 26 | 12 | NA | NA | NA | 21 | 48 | NA | NA | NA | NA | NA | NA | NA | NA |
| Mazine/2022 | 0 | NA | 0 | NA | NA | 1 | NA | 1 | NA | NA | 4 | NA | 2 | NA | NA | 3 | NA | 5 | NA | NA | 11 | NA | 63 | NA | NA | 10 | NA | 27 | NA | NA | NA | NA | NA | NA | NA |
| Mokhles/2011 | NA | NA | NA | NA | NA | NA | NA | NA | NA | NA | NA | NA | NA | NA | NA | NA | NA | NA | NA | NA | 8 | 0 | NA | NA | NA | 7 | 5 | NA | NA | NA | NA | NA | NA | NA | NA |
| Polito/2021/ | 1 | NA | NA | NA | 0 | NA | NA | NA | NA | NA | NA | NA | NA | NA | NA | NA | NA | NA | NA | NA | 1 | NA | NA | NA | 3 | 1 | NA | NA | NA | 2 | NA | NA | NA | NA | NA |
| Santini/1997 | 0 | NA | NA | 0 | NA | NA | NA | NA | NA | NA | NA | NA | NA | NA | NA | 1 | NA | NA | 1 | NA | NA | NA | NA | NA | NA | 0 | NA | NA | 0 | NA | NA | NA | NA | NA | NA |
| Sharabiani/2016 | 8 | 11 | 4 | 1 | NA | NA | NA | NA | NA | NA | NA | NA | NA | NA | NA | NA | NA | NA | NA | NA | 38 | 46 | 41 | 21 | NA | 19 | 53 | 12 | 3 | NA | NA | NA | NA | NA | NA |
| Zacek/2016 | 0 | 0 | NA | NA | NA | NA | NA | NA | NA | NA | NA | NA | NA | NA | NA | NA | NA | NA | NA | NA | NA | NA | NA | NA | NA | NA | NA | NA | NA | NA | NA | NA | NA | NA | NA |

**Supplementary Table 15.** References of Included Studies.

1. Aicher D, Holz A, Feldner S, Köllner V, Schäfers HJ. Quality of life after aortic valve surgery: replacement versus reconstruction. *J Thorac Cardiovasc Surg*. 2011;142(2):e19-24. doi:10.1016/j.jtcvs.2011.02.006

2. Akhyari P, Bara C, Kofidis T, Khaladj N, Haverich A, Klima U. Aortic root and ascending aortic replacement. *Int Heart J*. 2009;50(1):47-57. doi:10.1536/ihj.50.47

3. Andreas M, Wiedemann D, Seebacher G, et al. The Ross procedure offers excellent survival compared with mechanical aortic valve replacement in a real-world setting. *Eur J Cardiothorac Surg*. 2014;46(3):409-413; discussion 413-414. doi:10.1093/ejcts/ezt663

4. Basude S, Trinder J, Caputo M, Curtis SL. Pregnancy outcome and follow-up cardiac outcome in women with aortic valve replacement. *Obstet Med*. 2014;7(1):29-33. doi:10.1177/1753495X13514382

5. Bouhout I, Noly PE, Ghoneim A, et al. Is the Ross procedure a riskier operation? Perioperative outcome comparison with mechanical aortic valve replacement in a propensity-matched cohort. *Interact Cardiovasc Thorac Surg*. 2017;24(1):41-47. doi:10.1093/icvts/ivw325

6. Buratto E, Shi WY, Wynne R, et al. Improved Survival After the Ross Procedure Compared With Mechanical Aortic Valve Replacement. *J Am Coll Cardiol*. 2018;71(12):1337-1344. doi:10.1016/j.jacc.2018.01.048

7. Carr-White GS, Kon M, Koh TW, et al. Right ventricular function after pulmonary autograft replacement of the aortic valve. *Circulation*. 1999;100(19 Suppl):II36-41. doi:10.1161/01.cir.100.suppl_2.ii-36

8. Choudhary SK, Mathur A, Chander H, et al. Aortic valve replacement with biological substitute. *J Card Surg*. 1998;13(1):1-8; discussion 9-10. doi:10.1111/j.1540-8191.1998.tb01045.x

9. da Costa F, Haggi H, Pinton R, Lenke W, Adam E, Costa IS. Rest and exercise hemodynamics after the Ross procedure: an echocardiographic study. *J Card Surg*. 1998;13(3):177-185. doi:10.1111/j.1540-8191.1998.tb01258.x

10. Dagenais F, Cartier P, Voisine P, et al. Which biologic valve should we select for the 45- to 65-year-old age group requiring aortic valve replacement? *J Thorac Cardiovasc Surg*. 2005;129(5):1041-1049. doi:10.1016/j.jtcvs.2004.10.041

11. Doss M, Wood JP, Kiessling AH, Moritz A. Comparative evaluation of left ventricular mass regression after aortic valve replacement: a prospective randomized analysis. *J Cardiothorac Surg*. 2011;6:136. doi:10.1186/1749-8090-6-136

12. Doss M, Wood JP, Martens S, Wimmer-Greinecker G, Moritz A. Do pulmonary autografts provide better outcomes than mechanical valves? A prospective randomized trial. *Ann Thorac Surg*. 2005;80(6):2194-2198. doi:10.1016/j.athoracsur.2005.06.006

13. El-Hamamsy I, Eryigit Z, Stevens LM, et al. Long-term outcomes after autograft versus homograft aortic root replacement in adults with aortic valve disease: a randomised controlled trial. *Lancet*. 2010;376(9740):524-531. doi:10.1016/S0140-6736(10)60828-8

14. El-Hamamsy I, Toyoda N, Itagaki S, et al. Propensity-Matched Comparison of the Ross Procedure and Prosthetic Aortic Valve Replacement in Adults. *J Am Coll Cardiol*. 2022;79(8):805-815. doi:10.1016/j.jacc.2021.11.057

15. Gofus J, Fila P, Drabkova S, et al. Ross procedure provides survival benefit over mechanical valve in adults: a propensity-matched nationwide analysis. *Eur J Cardiothorac Surg*. 2022;61(6):1357-1365. doi:10.1093/ejcts/ezac013

16. Grocott-Mason RM, Lund O, Elwidaa H, et al. Long-term results after aortic valve replacement in patients with congestive heart failure. Homografts vs prosthetic valves. *Eur Heart J*. 2000;21(20):1698-1707. doi:10.1053/euhj.1999.2040

17. Hanke T, Charitos EI, Paarmann H, Stierle U, Sievers HH. Haemodynamic performance of a new pericardial aortic bioprosthesis during exercise and recovery: comparison with pulmonary autograft, stentless aortic bioprosthesis and healthy control groups. *Eur J Cardiothorac Surg*. 2013;44(4):e295-301. doi:10.1093/ejcts/ezt367

18. Heuvelman HJ, Arabkhani B, Cornette JMJ, et al. Pregnancy outcomes in women with aortic valve substitutes. *Am J Cardiol*. 2013;111(3):382-387. doi:10.1016/j.amjcard.2012.09.035

19. Jaggers J, Harrison JK, Bashore TM, Davis RD, Glower DD, Ungerleider RM. The Ross procedure: shorter hospital stay, decreased morbidity, and cost effective. *Ann Thorac Surg*. 1998;65(6):1553-1557; discussion 1557-1558. doi:10.1016/s0003-4975(98)00288-4

20. Klieverik LMA, Bekkers JA, Roos JW, et al. Autograft or allograft aortic valve replacement in young adult patients with congenital aortic valve disease. *Eur Heart J*. 2008;29(11):1446-1453. doi:10.1093/eurheartj/ehm589

21. Knott-Craig CJ, Elkins RC, Santangelo KL, McCue C, Lane MM. Aortic valve replacement: comparison of late survival between autografts and homografts. *Ann Thorac Surg*. 2000;69(5):1327-1332. doi:10.1016/s0003-4975(00)01212-1

22. Laforest I, Dumesnil JG, Briand M, Cartier PC, Pibarot P. Hemodynamic performance at rest and during exercise after aortic valve replacement: comparison of pulmonary autografts versus aortic homografts. *Circulation*. 2002;106(12 Suppl 1):I57-I62.

23. Mazine A, David TE, Rao V, et al. Long-Term Outcomes of the Ross Procedure Versus Mechanical Aortic Valve Replacement: Propensity-Matched Cohort Study. *Circulation*. 2016;134(8):576-585. doi:10.1161/CIRCULATIONAHA.116.022800

24. Mazine A, David TE, Stoklosa K, Chung J, Lafreniere-Roula M, Ouzounian M. Improved Outcomes Following the Ross Procedure Compared With Bioprosthetic Aortic Valve Replacement. *J Am Coll Cardiol*. 2022;79(10):993-1005. doi:10.1016/j.jacc.2021.12.026

25. Mokhles MM, Körtke H, Stierle U, et al. Survival comparison of the Ross procedure and mechanical valve replacement with optimal self-management anticoagulation therapy: propensity-matched cohort study. *Circulation*. 2011;123(1):31-38. doi:10.1161/CIRCULATIONAHA.110.947341

26. Nötzold A, Hüppe M, Schmidtke C, Blömer P, Uhlig T, Sievers HH. Quality of life in aortic valve replacement: pulmonary autografts versus mechanical prostheses. *J Am Coll Cardiol*. 2001;37(7):1963-1966. doi:10.1016/s0735-1097(01)01267-0

27. Pibarot P, Dumesnil JG, Briand M, Laforest I, Cartier P. Hemodynamic performance during maximum exercise in adult patients with the ross operation and comparison with normal controls and patients with aortic bioprostheses. *Am J Cardiol*. 2000;86(9):982-988. doi:10.1016/s0002-9149(00)01134-6

28. Polito A, Albanese SB, Cetrano E, et al. Aortic Valve Neocuspidalization May Be a Viable Alternative to Ross Operation in Pediatric Patients. *Pediatr Cardiol*. 2021;42(3):668-675. doi:10.1007/s00246-020-02528-3

29. Santini F, Dyke C, Edwards S, et al. Pulmonary autograft versus homograft replacement of the aortic valve: a prospective randomized trial. *J Thorac Cardiovasc Surg*. 1997;113(5):894-899; discussion 899-900. doi:10.1016/S0022-5223(97)70262-9

30. Schmidtke C, Hüppe M, Berndt S, Nötzold A, Sievers HH. [Quality of life after aortic valve replacement. Self-management or conventional anticoagulation therapy after mechanical valve replacement plus pulmonary autograft]. *Z Kardiol*. 2001;90(11):860-866. doi:10.1007/s003920170084

31. Sharabiani MTA, Dorobantu DM, Mahani AS, et al. Aortic Valve Replacement and the Ross Operation in Children and Young Adults. *J Am Coll Cardiol*. 2016;67(24):2858-2870. doi:10.1016/j.jacc.2016.04.021

32. von Knobelsdorff-Brenkenhoff F, Trauzeddel RF, Barker AJ, Gruettner H, Markl M, Schulz-Menger J. Blood flow characteristics in the ascending aorta after aortic valve replacement--a pilot study using 4D-flow MRI. *Int J Cardiol*. 2014;170(3):426-433. doi:10.1016/j.ijcard.2013.11.034

33. Wang A, Jaggers J, Ungerleider RM, Lim CS, Ryan T. Exercise echocardiographic comparison of pulmonary autograft and aortic homograft replacements for aortic valve disease in adults. *J Heart Valve Dis*. 2003;12(2):202-208.

34. Zacek P, Holubec T, Vobornik M, et al. Quality of life after aortic valve repair is similar to Ross patients and superior to mechanical valve replacement: a cross-sectional study. *BMC Cardiovasc Disord*. 2016;16:63. doi:10.1186/s12872-016-0236-0
